# The Activation of *Phytophthora* Effector Avr3b by Plant Cyclophilin is Required for the Nudix Hydrolase Activity of Avr3b

**DOI:** 10.1371/journal.ppat.1005139

**Published:** 2015-08-28

**Authors:** Guanghui Kong, Yao Zhao, Maofeng Jing, Jie Huang, Jin Yang, Yeqiang Xia, Liang Kong, Wenwu Ye, Qin Xiong, Yongli Qiao, Suomeng Dong, Wenbo Ma, Yuanchao Wang

**Affiliations:** 1 College of Plant Protection, Nanjing Agricultural University, Nanjing, China; 2 Department of Plant Pathology and Microbiology, University of California, Riverside, Riverside, California, United States of America; 3 National Key Facility for Crop Gene Resources and Genetic Improvement, Institute of Crop Science, Chinese Academy of Agricultural Sciences, Beijing, China; Scottish Crop Research Institute, UNITED KINGDOM

## Abstract

Plant pathogens secrete an arsenal of effector proteins to impair host immunity. Some effectors possess enzymatic activities that can modify their host targets. Previously, we demonstrated that a *Phytophthora sojae* RXLR effector Avr3b acts as a Nudix hydrolase when expressed *in planta*; and this enzymatic activity is required for full virulence of *P*. *sojae* strain P6497 in soybean (*Glycine max*). Interestingly, recombinant Avr3b produced by *E*. *coli* does not have the hydrolase activity unless it was incubated with plant protein extracts. Here, we report the activation of Avr3b by a prolyl-peptidyl isomerase (PPIase), cyclophilin, in plant cells. Avr3b directly interacts with soybean cyclophilin GmCYP1, which activates the hydrolase activity of Avr3b in a PPIase activity-dependent manner. Avr3b contains a putative Glycine-Proline (GP) motif; which is known to confer cyclophilin-binding in other protein substrates. Substitution of the Proline (P132) in the putative GP motif impaired the interaction of Avr3b with GmCYP1; as a result, the mutant Avr3b^P132A^ can no longer be activated by GmCYP1, and is also unable to promote *Phytophthora* infection. Avr3b elicits hypersensitive response (HR) in soybean cultivars producing the resistance protein Rps3b, but Avr3b^P132A^ lost its ability to trigger HR. Furthermore, silencing of *GmCYP1* rendered reduced cell death triggered by Avr3b, suggesting that GmCYP1-mediated Avr3b maturation is also required for Rps3b recognition. Finally, cyclophilins of *Nicotiana benthamiana* can also interact with Avr3b and activate its enzymatic activity. Overall, our results demonstrate that cyclophilin is a “helper” that activates the enzymatic activity of Avr3b after it is delivered into plant cells; as such, cyclophilin is required for the avirulence and virulence functions of Avr3b.

## Author Summary

*Phytophthora sojae*, an oomycete pathogen that causes the *Phytophthora* root and stem rot disease of soybean, delivers variety of effectors into host cell to reprogram host immunity. Genome sequencing uncovers that *P*. *sojae* genome encode several hundreds of effector genes. However, the mode of action of most of the *P*. *sojae* effectors remains unknown. The investigation of effector-interacting proteins provides opportunities to better understand the pathogenesis mechanism of the pathogen and the defense mechanism of the host plants. Previously, we reported that *P*. *sojae* avirulence effector Avr3b modulates plant immunity through its Nudix hydrolase activity. Interestingly, the enzymatic activity is required for its virulence but not required for recognition by resistant gene. In this study, we identified soybean cyclophilin protein GmCYP1 as an Avr3b interactor. The enzymatic activity of GmCYP1 is required for the maturation Avr3b, which is directly related to both virulence and avirulence functions of Avr3b. This work provides a novel insight into how *Phytophthora* pathogens recruit host proteins to activate the enzymatic activity of effectors in order to gain successful infection.

## Introduction

Plants have two layers of defense in response to microbial pathogen attacks [1]. Pattern-triggered immunity (PTI) is dependent on the recognition of microbe- or pathogen-associated molecular patterns (MAMPs or PAMPs) by surface-localized pattern recognition receptors (PRRs) [2]. The second defense system is effector-triggered immunity (ETI), which relies on the perception of pathogen effectors [1]. ETI produces a faster and stronger resistance response than PTI and often induces localized cell death in the infected area [1,3]. Effectors are usually rapidly evolving in pathogens due to dynamic and sometimes opposite evolutionary forces during the arms race with the hosts [4].

Plant pathogens secrete a diverse repertoire of effectors to overcome plant immunity and enable infection [5,6]; however, the biochemical functions of most effectors, especially those produced by eukaryotic pathogens, remain largely unknown. To our knowledge, only a few effectors produced by phytopathogenic oomycetes and fungi have clear biochemical functions and manipulate their host targets by enzymatic activity. For example, Cmu1 produced by the fungal pathogen *Ustilago maydis* has chorismate mutase activity and affects salicylic acid (SA) levels in maize cells [7]. Similarly, PsIsc1 from the oomycete pathogen *Phytophthora sojae* and VdIsc1 from the fungal pathogen *Verticillium dahliae* also suppress SA-mediated immunity through their isochorismatase activity [8].

*Phytophthora* is a group of notorious plant pathogens that infect a wide range of crop, vegetable, horticultural, pasture plants, and forest trees. Like many other plant pathogens, *Phytophthora* secretes effectors to gain virulence. The functions of an increasing number of *Phytophthora* effectors, particularly the RXLR (arginine, any, leucine, arginine) effectors that can be delivered into host cell, have recently been reported [5]. Many RxLR effectors can suppress PTI and ETI. For example, various effectors from the soybean (*Glycine max*) pathogen *P*. *sojae*, including Avr1b, Avr3b and Avr1d, are able to abolish cell death triggered by other effectors and/or PAMP in soybean or *Nicotiana benthamiana* [9–12]. Functional analysis of thirty-three RXLR effectors from the potato and tomato pathogen *Phytophthora infestans* revealed eight of them that could suppress PTI triggered by the bacterial flagellin peptide flg22 in tomato protoplasts [13].

Further investigations have revealed the molecular basis by which RXLR effectors suppress plant immunity. For example, PexRD2 perturbs plant immunity-related signaling by interacting with the kinase domain of MAPKKKε and reducing the accumulation of phosphorylated MAPK [14]. AVRblb2 interacts with host papain-like cysteine protease C14, which is a positive regulator of plant immunity, and prevents its secretion into apoplast [15]. Pi03192 targets NAC transcription factor NTP1 and NTP2 and prevents *Phytophthora* culture filtrate triggered re-localization of these proteins from the endoplasmic reticulum (ER) into the nucleus [16]. Another important discovery is that *Phytophthora* RXLR effectors could target RNA silencing pathway. *Phytophthora* suppressors of RNA silencing 1 and 2 (PSR1 and PSR2, respectively) of *P*. *sojae* are powerful suppressors of RNA silencing in plants and enhance plant susceptibility to *Phytophthora* by inhibiting the biogenesis of small RNAs [17,18].

We previously demonstrated that the *P*. *sojae* RXLR effector Avr3b possesses the Nudix hydrolase activity and contributes to full virulence of *P*. *sojae* [19]. Nudix hydrolases hydrolyze a wide range of organic pyrophosphates, including nucleoside di- and triphosphates, dinucleoside and diphosphoinositol polyphosphates, nucleotide sugars and RNA caps, which may be toxic or have regulatory roles [20]. As such, Nudix hydrolases play a key role in signaling, house-keeping processes and maintaining cellular homeostasis [21]. In particular, *Arabidopsis thaliana* Nudix protein AtNUDT7 was found to be a negative regulator of the basal defense response. Loss-of-function mutation of AtNUDT7 results in higher levels of salicylic acid (SA) and constitutive expression of defense-related genes, and thereby enhanced resistance to the bacterial pathogen *Pseudomonas syringae* and the oomycete pathogen *Hyaloperonospora arabidopsidis* (*Hpa*) [22–25]. Besides Avr3b, Nudix effector CtNUDIX from the fungal pathogen *Colletotrichum truncatum* is exclusively expressed during the late biotrophic phase and elicits a HR-like cell death in tobacco leaves, suggesting that CtNUDIX may signal the transition from biotrophy to necrotrophy [26]. In addition, Hpx26 produced by the bacterial pathogen *Ralstonia solanacearu*m shares homology with known Nudix hydrolase [27]. So far, the biochemical functions of CtNUDIX and Hpx26 are unclear.

Cyclophilin (CYP) possesses peptidyl-prolyl *cis*-*trans* isomerase (PPIase) activity and catalyzes the isomerization of prolyl bonds in proteins [28,29]. CYP family members are involved in diverse aspects of cellular physiology including transcription, immune response, mitochondrial function, cell death, and chemotaxis [30–32]. Interestingly, CYP plays a critical role in the activation of bacterial effectors and the life cycle of viruses, especially hepatitis C virus (HCV) and human immunodeficiency virus (HIV) [30,33,34]. For example, AvrRpt2 from *P*. *syringae* is delivered into the plant cell as an inactive form and subsequently activated by host cyclophilin ROC1 (Rotamase CYP1) [33]. During HCV infection of animal cells, cyclophilin B interacts with the HCV RNA polymerase NS5B and functions as a stimulatory regulator of NS5B in HCV replication machinery [35].

The Nudix hydrolase activity of Avr3b is important for *P*. *sojae* infection. However, recombinant Avr3b proteins produced in *Escherichia coli* had no hydrolase activity unless it was incubated with plant extracts, suggesting that unknown plant factors is required for the activation of Avr3b in host cells. Here, we identified the soybean cyclophilin protein GmCYP1 as an Avr3b-interacting protein and showed that its PPIase activity is responsible for the activation of Avr3b enzymatic activity. We further identified the Pro132 residue in a potential GP motif of Avr3b as a key residue involved in Avr3b-GmCYP1 interaction. The mutant Avr3b^P132A^, no longer possessing the hydrolase activity, is also abolished for its virulence activity in *N*. *benthamiana* as well as HR-triggering activity in a resistant soybean cultivar that produces the cognate resistance (R) protein of Avr3b, Rps3b. Moreover, application of a PPIase inhibitor, cyclosporine A (CsA), or silencing of *GmCYP1* greatly reduced the cell death triggered by Avr3b in soybean, suggesting that GmCYP1 is required for the maturation of Avr3b as a Nudix hydrolase and therefore playing an essential role in the virulence and avirulence activities of Avr3b *in planta*.

## Results

### Avr3b is activated *in planta*

**Fig 1:**
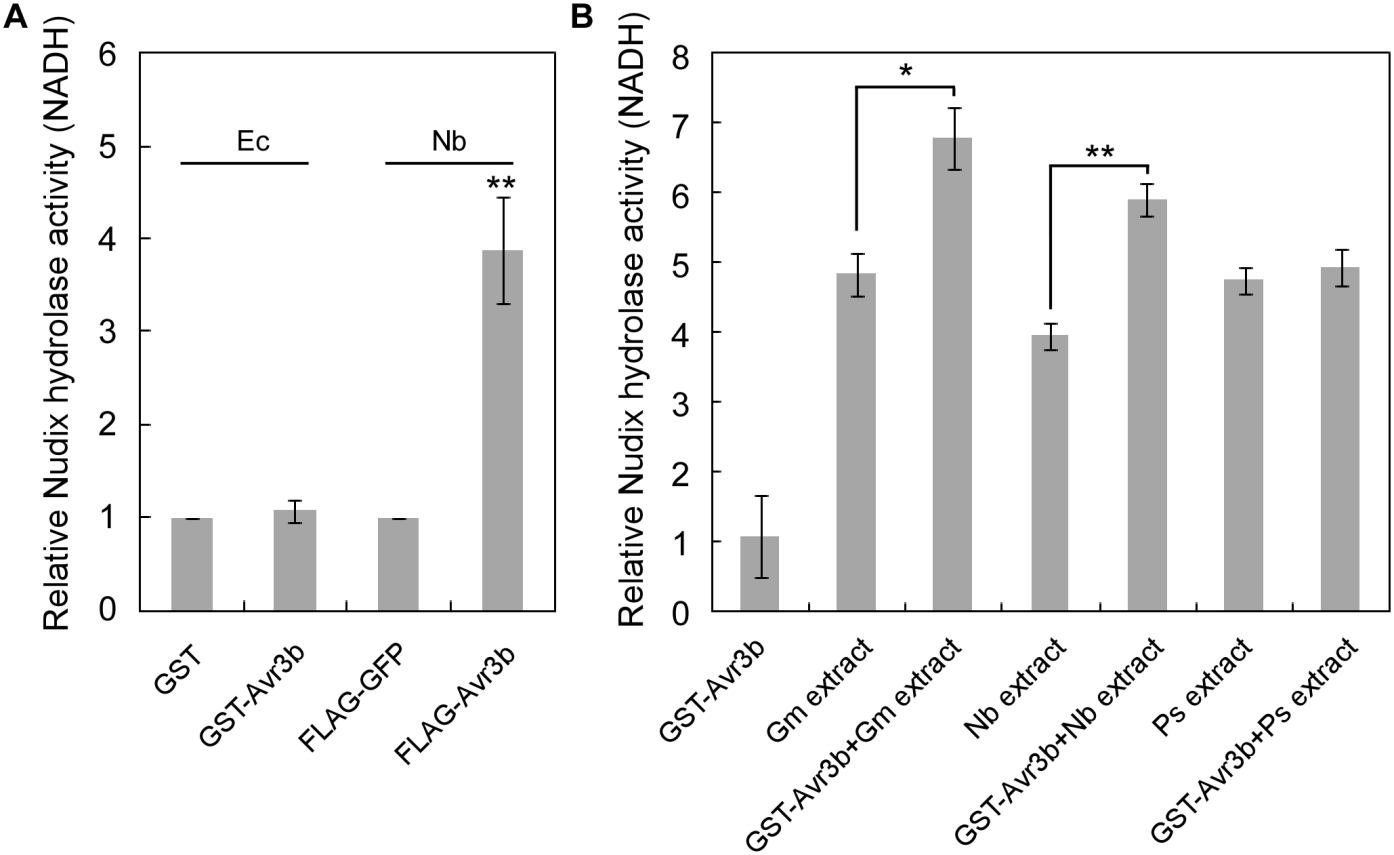
The enzymatic activity of Avr3b is activated by plant factors. (**A**) Avr3b only has Nudix hydrolase activity when produced in plants. Avr3b was ectopically expressed in *E*. *coli* (Ec) or *N*. *benthamiana* (Nb). Relative Nudix hydrolase activity was calculated by comparing enzymatic activity of purified GST-Avr3b with GST produced in *E*. *coli* or purified FLAG-Avr3b with FLAG-GFP in *N*. *benthamiana*. NADH was applied as substrate in Nudix hydrolase activity assays. Means and standard errors from three replicates are shown. ** representing *t* test *P* < 0.01. (**B**) *E*. *coli* produced Avr3b protein can be activated by incubation with plant protein extracts. Recombinant GST-Avr3b protein was purified from *E*. *coli*. The recombinant protein (2 μg) was incubated with 100 μg dialyzed soybean (*Glycine max*, Gm) extract, 100 μg dialyzed *N*. *benthamiana* extract, or 100 μg dialyzed *P*. *sojae* (Ps) mycelium extract at 25°C for 15 hours. The Nudix hydrolase activity was then determined using FLAG-GFP as a control. Means and standard errors from three measurements are shown. ** or *, representing *t* test *P* < 0.01 or 0.05, respectively.

Previous study demonstrated that Avr3b is a functional Nudix hydrolase, and this enzymatic activity is essential for the virulence function of Avr3b [19]. To further elucidate the enzymatic activity of Avr3b, we expressed the recombinant GST-Avr3b protein in *N*. *benthamiana* and *E*. *coli*, respectively. Interestingly, unlike Avr3b expressed in *N*. *benthamiana*, which exhibited clear Nudix hydrolase activity, purified Avr3b produced in *E*. *coli* could not hydrolyze the substrate NADH (Fig 1A). These results suggest that the enzymatic activity of Avr3b might need to be activated by some unknown factors from plants. To test this hypothesis, recombinant Avr3b produced by *E*. *coli* was incubated with dialyzed total protein extracts from *P*. *sojae*, soybean or *N*. *benthamiana* for 15 hours before the hydrolase activity was examined. Consistent with the previous results, addition of soybean or *N*. *benthamiana* extracts significantly promoted the hydrolase activity of Avr3b, while no change was observed after Avr3b was incubated with extracts from *P*. *sojae* mycelium (Fig 1B). These results suggest that Avr3b is likely in an inactive form in *P*. *sojae* and activated after it is delivered into plant cells by a plant factor(s).

### Avr3b interacts with plant cyclophilins

To investigate the underlying mechanism of Avr3b activation in plant cells, we performed yeast two-hybrid (Y2H) screens and identified plant proteins that associate with Avr3b using a soybean cDNA library. One protein that was repeatedly identified from three independent screens is a soybean cyclophilin protein GmCYP1 (Gm11g10480). We then focused our research on GmCYP1 because cyclophilin was shown to activate the cysteine protease activity of a bacterial effector AvrRpt2 in *Arabidopsis* [33,34].

**Fig 2:**
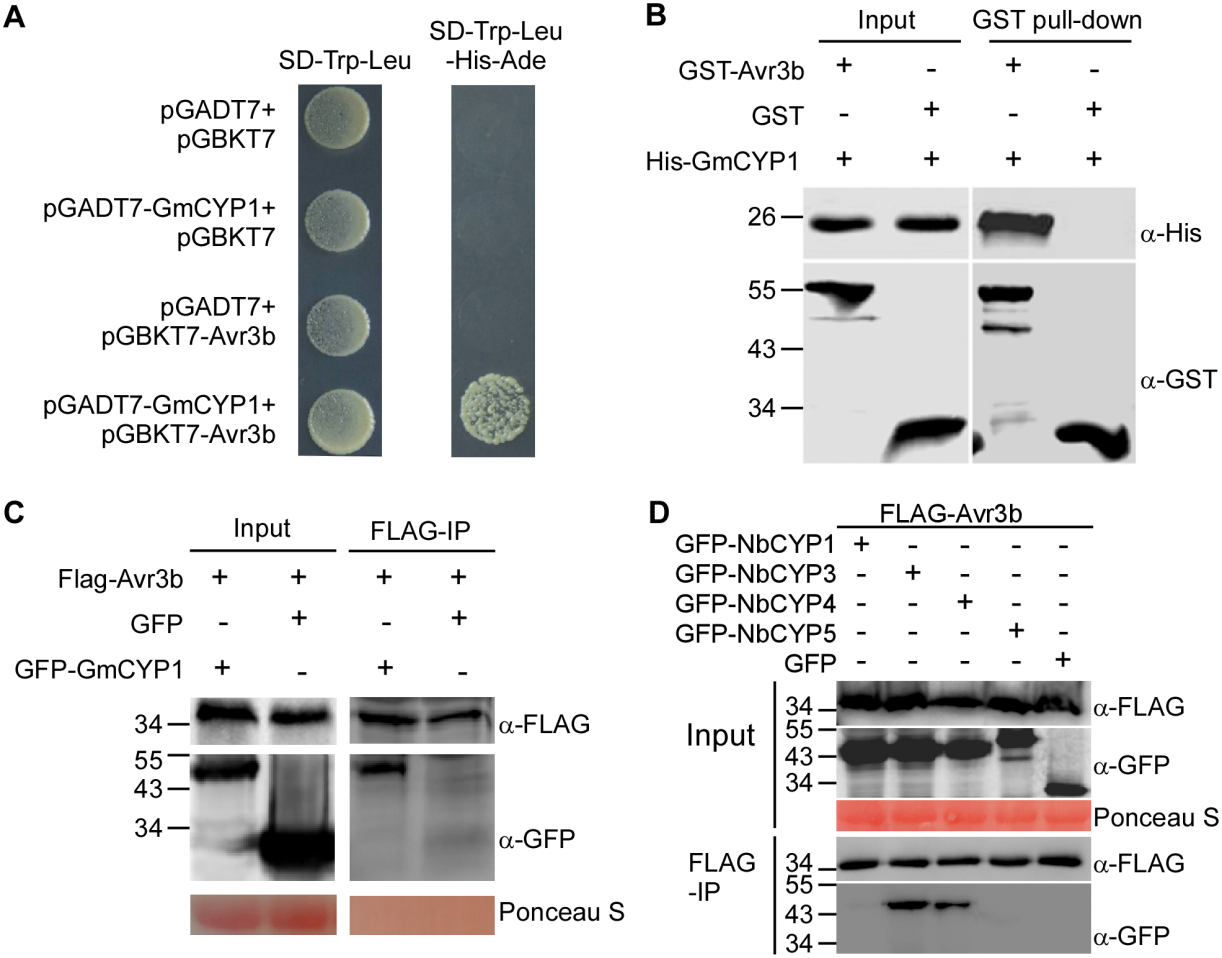
Avr3b interacts with plant cyclophilins. (**A**) Avr3b interacts with GmCYP1 in yeast. Avr3b and GmCYP1 were cloned into pGBKT7 and pGADT7 vectors, respectively. Yeast transformants were grown on SD/-Trp/-Leu (SD-2) or the selective medium SD/-Trp/-Leu/-His/-Ade (SD-4). The plates were photographed 2 days after inoculation. (**B**) Avr3b physically interacts with GmCYP1 in vitro. GST-Avr3b or GST bound resins were incubated with *E*. *coli* cell lysate containing His-GmCYP1. Co-precipitation of His-GmCYP1 with the GST-binding resins was examined by western blots using anti-His antibody before (Input) and after resins incubation (Pull-down). (**C**) Avr3b interacts with GmCYP1 in plant cells. FLAG-Avr3b was transiently expressed with either GFP-GmCYP1 or GFP in *N*. *benthamiana* leaves. FLAG-tagged Avr3b was immunoprecipitated by anti-FLAG M2 affinity gel from total plant extracts. Input controls (Input) and M2 gel binding proteins (IP) were analyzed by western blots using anti-FLAG or anti-GFP antibody. (**D**) Avr3b interacts with NbCYP3 and NbCYP4 in *N*. *benthamiana*. FLAG-Avr3b was transiently expressed with GFP-tagged protein fusions of NbCYPs or GFP in *N*. *benthamiana*. Immunoprecipitates pulled down using anti-FLAG M2 affinity gel from total protein extracts were immunoblotted with anti-FLAG or anti-GFP antibodies. IP, immunoprecipitate. These experiments were repeated three times with similar results.

The Avr3b-GmCYP1 interaction was validated using three independent assays. First, we cloned the full length cDNA sequence of *GmCYP1* into the Y2H vector pGADT7 and confirmed its association with Avr3b in yeast (Fig 2A). Secondly, we purified the recombinant GST-Avr3b, His-GmCYP1 proteins from *E*. *coli* and detected co-precipitation of His-GmCYP1 with GST-Avr3b in vitro, but not with GST, using glutathione resins (Fig 2B and S1 Fig). Finally, we confirmed the interaction between Avr3b and GmCYP1 in *N*. *benthamiana* using co-immunoprecipitation. FLAG-Avr3b was co-expressed with GFP-GmCYP1 or GFP in *N*. *benthamiana* leaves using *Agrobacterium*-mediated transient expression. Enrichment of GFP-GmCYP1 was detected in the FLAG-Avr3b precipitates using anti-FLAG affinity gel from total protein extracts (Fig 2C). Taken together, these experiments demonstrate that Avr3b directly interacts with GmCYP1 *in vitro* and *in vivo*.

Because Avr3b could be activated by protein extract of *N*. *benthamiana*, we hypothesized that cyclophilin proteins in *N*. *benthamiana* could interact with Avr3b and activate its Nudix hydrolase activity. To test this hypothesis, we examined whether Avr3b interacts with GmCYP1 homologs in *N*. *benthamiana*. A phylogenetic tree was constructed based on the conserved cyclophilin domain of cyclophilin proteins from *N*. *benthamiana*, soybean and *P*. *sojae* (S2 Fig). Five *N*. *benthamiana* cyclophilins (NbCYP1—NbCYP5) were selected as they share the highest (74%-94%) similarity to GmCYP1 in amino acid sequences. Among them, NbCYP1 (NbC26100015g0001) and NbCYP2 (NbS00003953g0001) are identical in their full-length amino acid sequences; NbCYP3 (NbS00044621g0001) and NbCYP4 (NbS00014422g0001) also share high identity (98% identity). These NbCYPs were then examined for their interactions with Avr3b. Using *in planta* co-immunoprecipitation assay, we were able to detect enrichment of NbCYP3 and NbCYP4, but not NbCYP1 or NbCYP5 (NbS00058430g0005), in the FLAG-Avr3b precipitates (Fig 2D). These results demonstrate that NbCYP3 and NbCYP4 can interact with Avr3b *in planta*.

Taken together, these results suggest that Avr3b associates with specific cyclophilin proteins in plant cells, such as GmCYP1 in soybean and NbCYP3 and NbCYP4 in *N*. *benthamiana*.

### Cyclophilin activates the hydrolase activity of Avr3b

**Fig 3:**
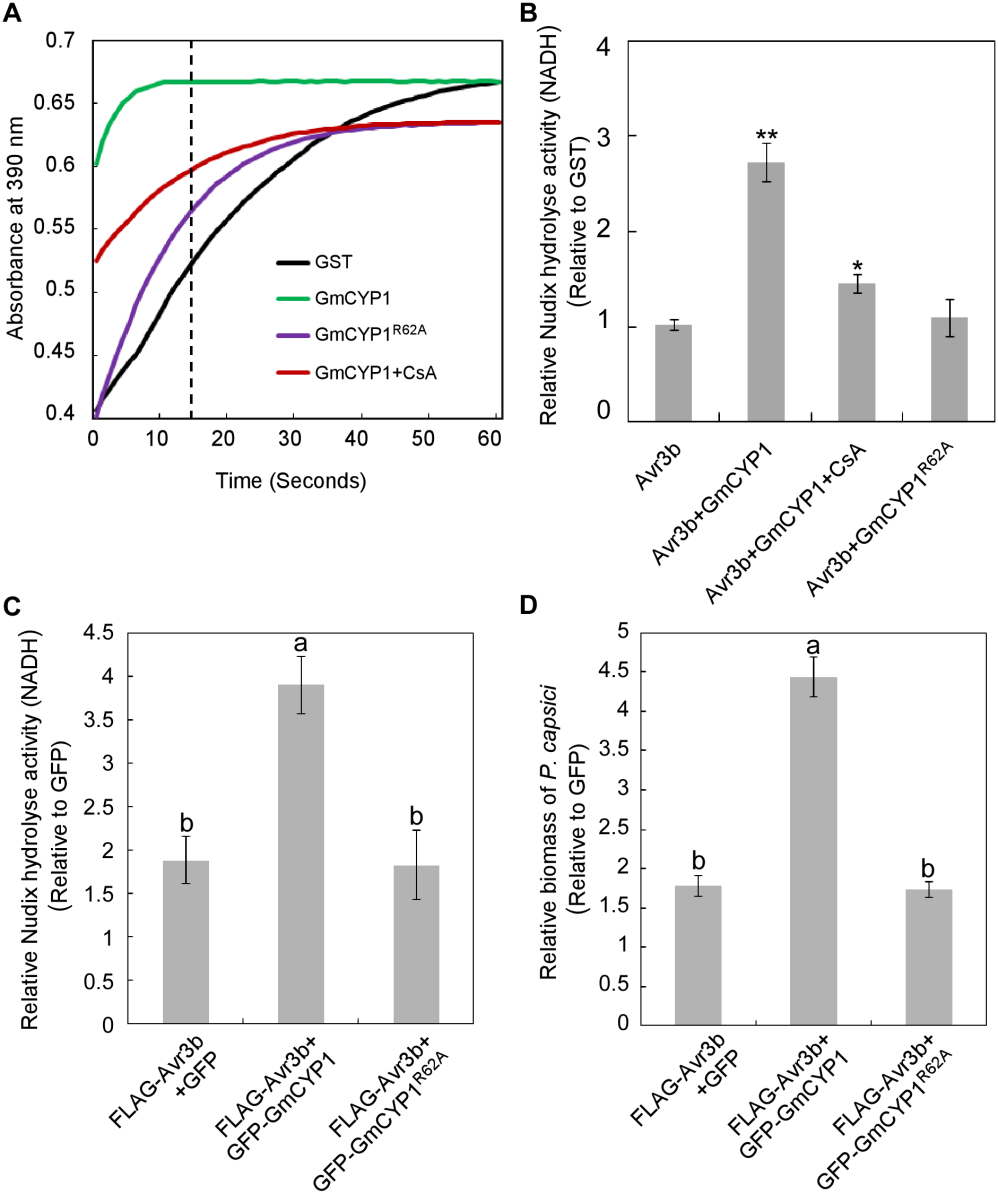
Activation of Avr3b is dependent on the PPIase activity of GmCYP1. (**A**) GmCYP1 possesses peptidyl-prolyl *cis-trans* isomerase (PPlase) activity. GmCYP1^R62A^ (PPIase-deficient mutant) was generated by site-directed mutagenesis. The PPIase enzymatic activity of GST, GmCYP1, and GmCYP1^R62A^ produced in *E*. *coli* was analyzed using a chymotrypsin-coupled assay. Enzyme activity can be assessed by analyzing the peak of the curve 15 s (dotted line indicated) after the adding of α-chymotrypsin. A higher absorbance in 390 nm indicates increased PPIase activity. 20 μM cyclosporine A (CsA) was added to the reaction when appropriate as a chemical inhibitor of the PPIas activity. Three independent replicates were performed with similar results. (**B**) Avr3b can be activated by GmCYP1 in vitro. GST-Avr3b proteins produced from *E*. *coli* were incubated with purified GmCYP1or GmCYP1^R62A^. CsA was supplemented to the reactions when appropriate. The Nudix hydrolase activity was then determined using GST as a control. Means and standard errors from three measurements are shown. ** or *, representing significantly different than the Avr3b *t* test *P* < 0.01 or 0.05, respectively. (**C**) GmCYP1 enhances the Nudix hydrolase activity of Avr3b *in planta*. Avr3b was expressed with GFP-GmCYP1, GFP-GmCYP1^R62A^ or GFP in *N*. *benthamiana* leaves using *Agrobacterium*-mediated transient expression and Nudix hydrolase activity was analyzed 48 hours post *Agro*-infiltration (hpi). Bars represent standard errors from four biological replicates. The same letter indicates no significant difference between values, and different letters indicate significant differences between values (P < 0.01, nonparametric Kruskal-Wallis test). (**D**) GmCYP1 enhances the virulence activity of Avr3b in *N*. *benthamiana*. Leaves expressing Avr3b with GFP-GmCYP1, GFP-GmCYP1^R62A^ or GFP were inoculated with zoospore suspension of *P*. *capsici* 48 hpi. DNA from *P*. *capsici* infected regions was isolated at 36 hours post *P*. *capsici* inoculation, and the biomass of *P*. *capsici* in infected tissues was determined using quantitative PCR comparing with GFP-expressing tissues. Bars represent standard errors from three biological replicates. The letters represent statistical significance (P < 0.01 nonparametric Kruskal-Wallis test).

Cyclophilins serve as protein folding catalysts by regulating *cis*-*trans* isomerization of prolyl bonds [36]. We then confirmed that GmCYP1 possesses the peptidyl-prolyl *cis-trans* isomerase (PPIase) activity by the chymotrypsin-coupled assay using N-Succinyl-Alanine-Proline-Phenylalanine-P-Nitroanilide (Suc-AAPF-pNA) as the alpha-chymotrypsin substrate [37]. In the presence of functional PPIase, the Suc-AAPF-pNA prolyl bond is more rapidly converted to the *trans* conformation, which can be cleaved by chymotrypsin, leading to the formation of a colored product 4-nitroaniline [38]. As such, in the presence of active PPIase, we would observe a rapid increase in absorbance at 390 nm. As expected, Recombinant His-GmCYP1 protein purified from *E*. *coli* showed clear PPIase activity compared to GST, which served as a negative control (Fig 3A). Based on previous studies, Arg62 of GmCYP1 might be essential for the PPIase activity [34,39], we constructed the mutant GmCYP1^R62A^, which indeed lost the PPIase activity but still interacted with Avr3b (Fig 3A and S3 Fig). In addition, the PPIase activity of GmCYP1 can be significantly inhibited in the presence of 20 μM cyclosporine A (CsA), a chemical inhibitor of cyclophilin (Fig 3A). These results demonstrate that GmCYP1 is a canonical cyclophilin with the PPIase activity.

Next, we examined whether Avr3b is activated by GmCYP1 via its PPIase activity. Purified GST-Avr3b proteins produced by *E*. *coli* were incubated with His-GmCYP1 or His-GmCYP1^R62A^ (S4 Fig). The Nudix hydrolase activity of GST-Avr3b was significantly enhanced after incubation with His-GmCYP1, but not with His-GmCYP1^R62A^ (Fig 3B and S1 Table). Consistently, activation of Avr3b Nudix hydrolase activity by GmCYP1 was significantly weakened in the presence of CsA (Fig 3B). These results suggest that Avr3b is activated by GmCYP1 in a manner that is dependent on PPIase activity.

To study the interaction between Avr3b and GmCYP1 *in planta*, Avr3b was co-expressed with GFP-GmCYP1, GFP-GmCYP1^R62A^ or GFP in *N*. *benthamiana* leaves. *N*. *benthamiana* leaves transiently expressing Avr3b and GmCYP1 showed a higher level of Nudix hydrolase activity (Fig 3C and S1 Table) and increased susceptibility to *P*. *capsici* (Fig 3D). This difference was not due to different protein expression levels of Avr3b as shown by western blots (S5 Fig). These results suggest that GmCYP1 can promote Avr3b maturation *in planta*.

### NbCYP3 and NbCYP4 activate Avr3b in *N*. *benthamiana*

Our previous experiments showed that NbCYP3 and NbCYP4 interact with Avr3b *in planta*, we next determined whether NbCYP3 and NbCYP4 can activate Avr3b processing. *NbCYP3* and *NbCYP4* were silenced in *N*. *benthamiana* by virus-induced gene silencing (VIGS) using Tobacco Rattle Virus (TRV)-based vectors. Because *NbCYP3* and *NbCYP4* are highly similar, our TRV:*NbCYP3* construct effectively silenced both genes, but the transcript levels of the homologous genes, *NbCYP1*, *NbCYP2*, and *NbCYP5*, remained unchanged (S6A Fig). These results confirmed that the silencing construct specifically knocked down the expression of the Avr3b-interacting *NbCYP3* and *NbCYP4*.

**Fig 4:**
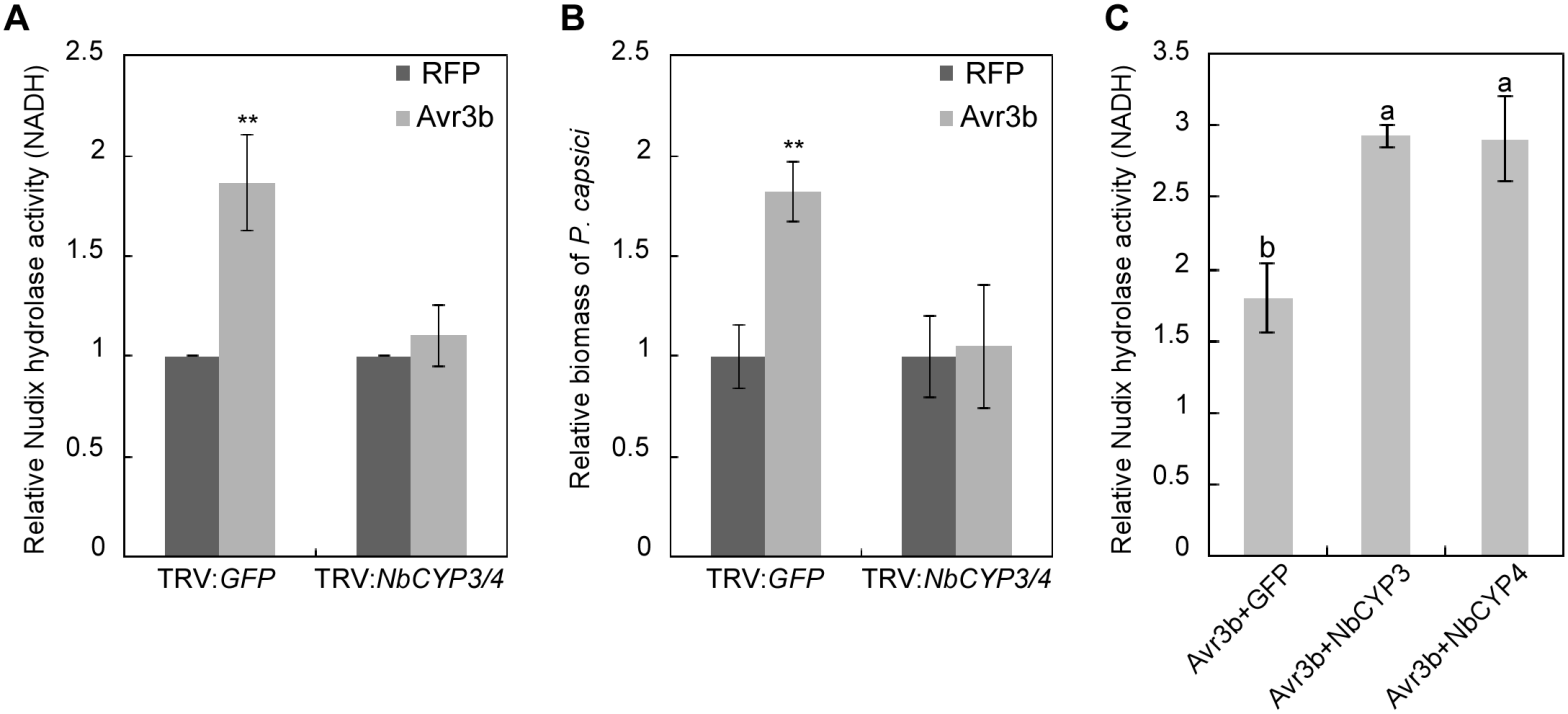
NbCYP3 and NbCYP4 are required for the Nudix hydrolase and the virulence activity of Avr3b in *N*. *benthamiana*. (**A**) NbCYP3/4-silenced *N*. *benthamiana* can not activate the Nudix hydrolase activity of Avr3b. Avr3b and RFP (as a negative control) were transiently expressed in *NbCYP3/4*-silenced *N*. *benthamiana* leaves. Total proteins were extracted at 48 hpi, and the Nudix hydrolase activity was measured using NADH as substrate. Means and standard errors from four measurements are shown. **, *t* test P<0.01. (**B**) NbCYP3 and NbCYP4 are required for the virulence activity of Avr3b in *N*. *benthamiana*. FLAG-RFP or FLAG-Avr3b proteins were transiently expressed in *N*. *benthamiana* leaves by *Agro*-infiltration. The leaves were inoculated with *P*. *capsici* at 48 hours post *Agro*-infiltration. Infection was determined using quantitative PCR to measure the ratios of *P*. *capsici* and *N*. *benthamiana* DNA at 36 hours post inoculation. Means and standard errors from four measurements are shown. ** representing *t* test *P* < 0.01. (**C**) NbCYP3 and NbCYP4 can enhance the Nudix hydrolase activity of Avr3b in *N*. *benthamiana*. FLAG-Avr3b was co-expressed in *N*. *benthamiana* leaves with GFP-NbCYP3 or GFP-NbCYP4 by *Agro*-infiltration method, and the Nudix hydrolase activity was measured using NADH as substrate comparing with *GFP*-expressing leaves. Bars represent standard errors from three biological replicates. The same letter indicates no significant difference between values, and different letters indicate significant differences between values (P < 0.01, nonparametric Kruskal-Wallis test).

Plants expressing the TRV:*NbCYP3/4* constructs did not show any marked phenotypic alterations compared to the control plants expressing TRV:*GFP* (S6B Fig). We then expressed Avr3b and RFP (as a control) in *NbCYP3/4*-silenced leaves through *Agro*-infiltration and analyzed the Nudix hydrolase activity. In contrast to the clear Nudix hydrolase activity detected in leaves expressing TRV:*GFP*, Avr3b did not exhibit any activity in *NbCYP3/4*- silenced leaves (Fig 4A). This is not due to different protein expression levels of Avr3b in these leaves (S6C Fig). Next, we infected these leaves with the *P*. *capsici*. In leaves that transiently expressed Avr3b, we observed significantly enlarged lesions in the TRV:*GFP* plants; however, this virulence activity of Avr3b was completely abolished in *NbCYP3/4*-silenced leaves (Fig 4B). From these experiments, we conclude that silencing of *NbCYP3* and *NbCYP4* disrupted the activation of Avr3b in *N*. *benthamiana*. To verify whether NbCYP3 and NbCYP4 are responsible for activating the Nudix hydrolase activity of Avr3b, we co-expressed Avr3b with NbCYP3, NbCYP4 or GFP in *N*. *benthamiana*. Over-expression of NbCYP3 and NbCYP4 in *N*. *benthamiana* led to further enhancement on the Nudix hydrolase activity of Avr3b (Fig 4C), supporting a role of NbCYP3 or NbCYP4 in Avr3b activation in *N*. *benthamiana*.

### PPIase activity of GmCYP1 is required for Avr3b-induced cell death in soybean

**Fig 5:**
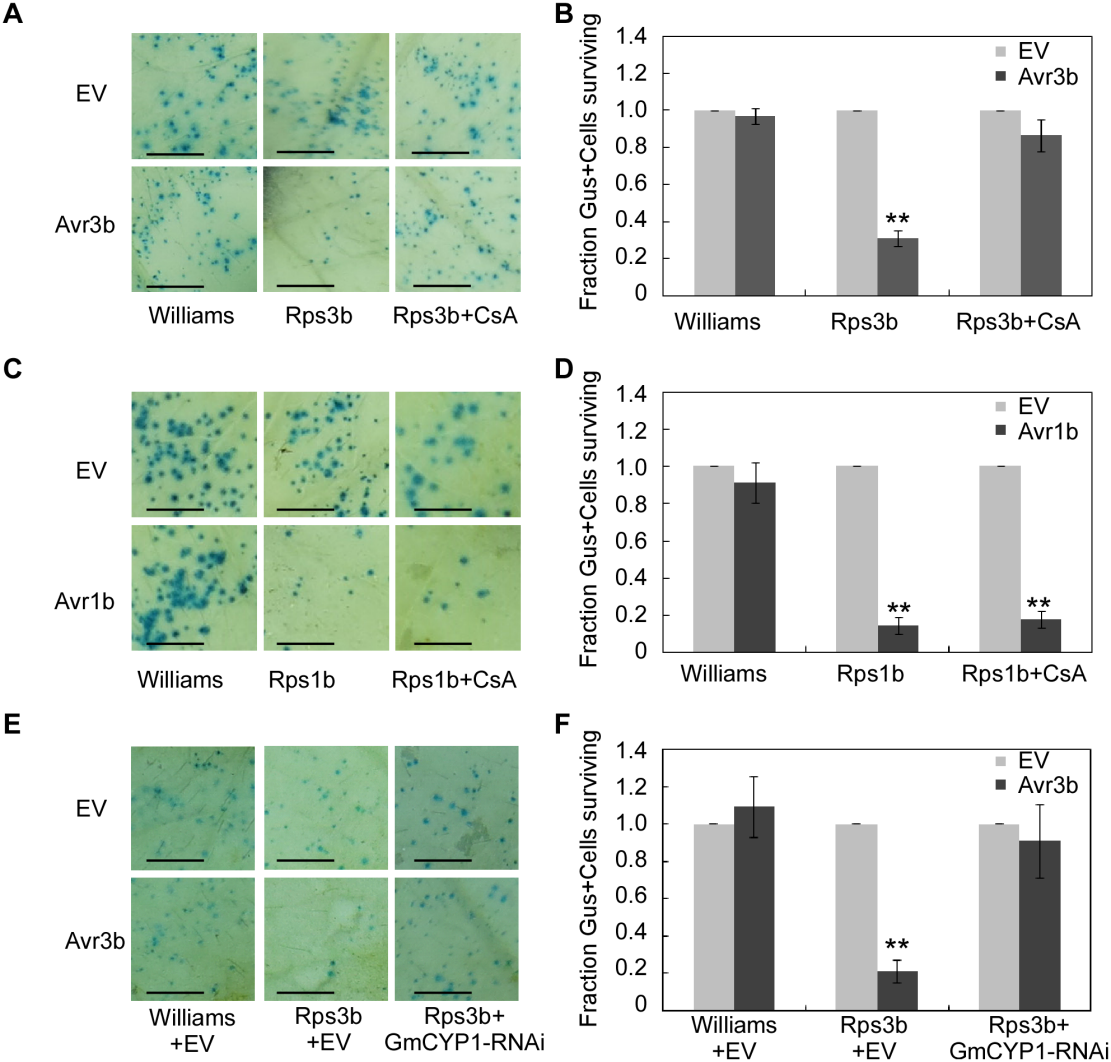
Avr3b recognition in soybean is dependent on the PPlase activity of GmCYP1. (**A, B**) CsA suppresses Avr3b-triggered cell death in soybean leaves. **(A)** Soybean cultivars Williams (rps3b) and PRX146-36 (Rps3b) were transformed by co-bombardment with a plasmid mixture consisting of a β-glucuronidase (GUS) expression vector and Avr3b or empty vector (EV). Soybean leaves were sprayed 20 μM CsA solution immediately after bombardment and incubated for 2 days in darkness at 28°C. The leaves were then stained using X-gluc. Scale bars, 3mm. (**B**) Percentage rate of GUS-positive blue spots following bombardment with Avr3b compared with EV. Bars represent standard errors from four independent replicates. **, *t* test *P*<0.01. (**C, D**) CsA could not suppress Avr1b-triggered cell death. (**C**) Avr1b was transiently expressed in soybean cultivars Williams (rps1b) and L77-1863 (Rps1b) by co-bombardment with GUS expression vector and Avr1b or EV. The leaves were incubated for 2 days in darkness at 28°C after bombardment, and then stained using X-gluc. Scale bars, 3mm. (**D**) Percentage rate of GUS-positive blue spots following bombardment with Avr1b compared with the empty vector. Bars represent standard errors from four independent replicates. **, *t* test *P*<0.01. (**E, F**) Avr3b failed to induce Rps3b-mediated cell death in *GmCYP1*-silenced soybean leaves. (**E**) Avr3b was delivered into leaves of soybean cultivars Williams (rps3b) and PRX146-36 (Rps3b) together with a *GmCYP1*-RNAi construct by co-bombardment. The leaves were incubated for 2 days in darkness at 28°C after bombardment, and then stained using X-gluc. Scale bars, 3mm. EV was used as a negative control. (**F**) Percentage rate of GUS-positive blue spots following bombardment with Avr3b compared with the EV in the presence of *GmCYP1*-RNAi. Bars represent standard errors from four independent replicates. **, *t* test *P*<0.01.

In soybean, resistance to *P*. *sojae* strains expressing *Avr3b* is conferred by the resistance protein Rps3b [19]. To test the role of GmCYP1 in Avr3b-induced cell death in resistant cultivars of soybean, CsA was used to suppress the PPIase activity in soybean leaves, which were subsequently tested for Avr3b-triggered HR. Our results showed that transient expression of Avr3b in Rps3b-producing soybean could induce cell death, which was readily blocked by spraying 20 μM CsA on the leaves (Fig 5A and 5B). To rule out the possibility that application of CsA inhibits general cell death in soybean, another *P*. *sojae* effector Avr1b was tested using soybean carrying its cognate R protein Rps1b [40]. The same CsA treatment did not disturb Avr1b-triggered cell death in Rps1b-producing soybean, indicating that CsA specifically suppressed Avr3b-triggered cell death (Fig 5C and 5D). These results suggest that the PPIase activity is important for the recognition of Avr3b, but not a general factor of ETI, in soybean.

To further investigate the role of GmCYP1 in Rps3b-mediated cell death in soybean, a hairpin RNAi construct targeting *GmCYP1* was introduced into soybean leaves together with Avr3b by co-bombardment (S7A Fig). Quantitative RT-PCR data showed that *GmCYP1* transcript level was significantly reduced in the delivery region two days after bombardment (S7B Fig). On the contrary, the transcript levels of another two soybean cyclophilin genes, *Gm04g00700* and *Gm06g00740*, both of which share high sequence identity (83% and 84% in full-length nucleic acid sequences respectively) with GmCYP1, were not reduced in leaves expressing the *GmCYP1*-RNAi construct (S7B Fig). The cell death assay indicated that Avr3b-triggered cell death was weakened in soybean when *GmCYP1* was silenced. (Fig 5E and 5F). Taken all the data together, our experiments demonstrate that GmCYP1 is required for the recognition of Avr3b by Rps3b, likely by modulating Avr3b protein structure via the PPIase activity.

### Pro132 of Avr3b is required for association with GmCYP1

**Fig 6:**
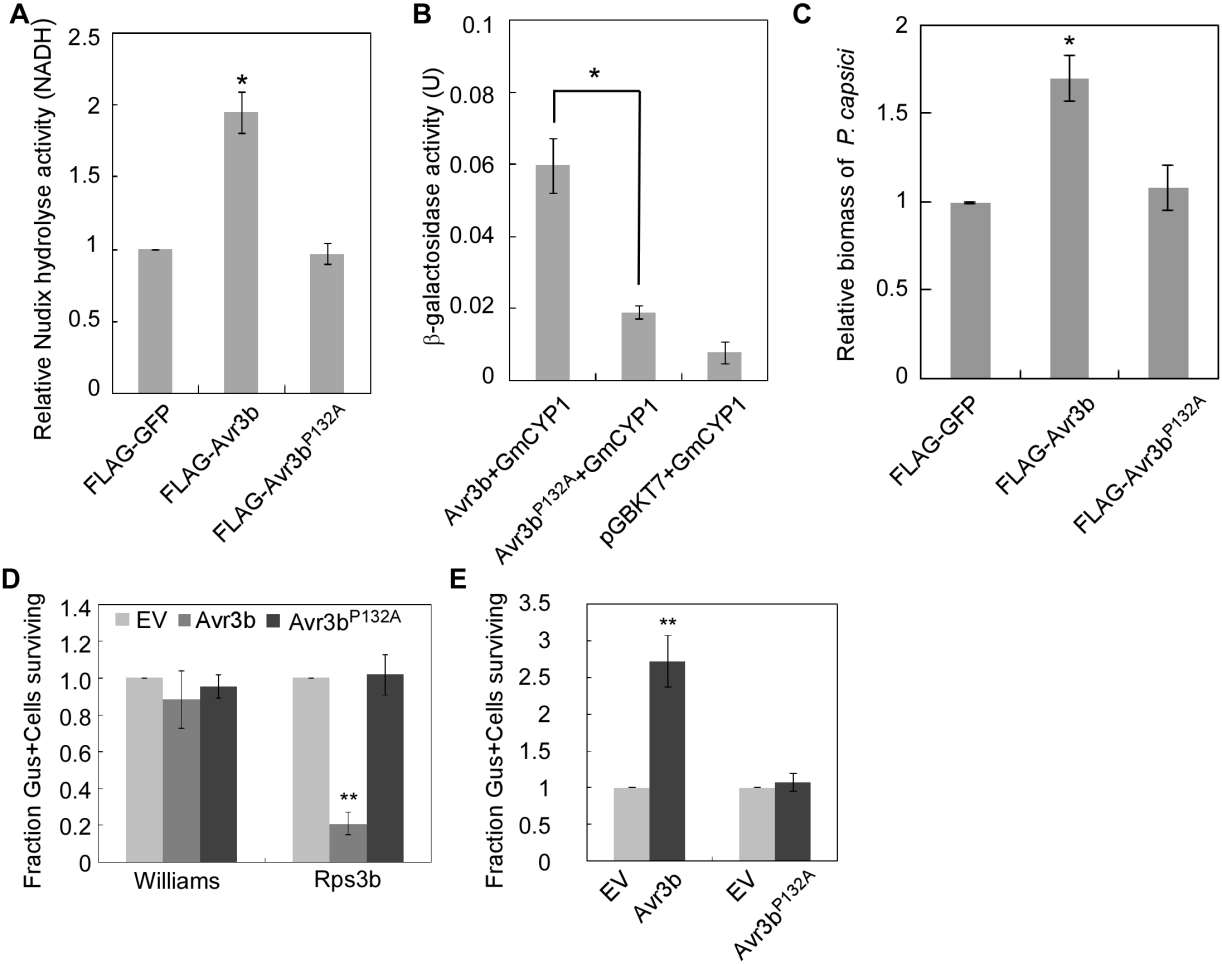
Proline132 of Avr3b plays a critical role in activation by cyclophilins. (**A**) Avr3b^P132A^ mutant can not be activated in *N*. *benthamiana*. FLAG-GFP, FLAG-Avr3b or FLAG-Avr3b^P132A^ were expressed in *N*. *benthamiana* leaves. Relative Nudix hydrolase activity was analyzed at 48 hpi. Bars represent standard errors from three independent replicates. * representing significantly different than FLAG-GFP (*t* test *P*<0.05). (**B**) Avr3b^P132A^ is impaired in its interaction with GmCYP1. Yeast cells were co-transformed with pGADT7-GmCYP1 and pGBKT7 carrying Avr3b or Avr3b^P132A^. Transformants were grown on a selective medium.β-galactosidase activity in yeast cells was measured to determine the Avr3b-GmCYP1 interaction. Bars represent standard errors from three independent replicates. *, *t* test *P*<0.05. (**C**) The P132A mutation of Avr3b resulted in loss of virulence activity. FLAG-GFP, FLAG-Avr3b or FLAG-Avr3b^P132A^ proteins were transiently expressed in *N*. *benthamiana* leaves by *Agro*-infiltration. The leaves were inoculated with *P*. *capsici* at 48 hours post *Agro*-infiltration. Biomass of *P*. *capsici* was determined at 36 hours post inoculation by quantitative PCR. Bars represent standard errors from three independent replicates. *, *t* test *P*<0.05. (**D**) Avr3b^P132A^ failed to induce Rps3b-mediated cell death in soybean. Percentage rate of β-glucuronidase (GUS)-positive blue spots following bombardment with Avr3b^P132A^ compared with the EV. Bars represent standard errors from four independent replicates. **, *t* test *P*<0.01. (**E**) Avr3b^P132A^ could not suppress Avr1b-triggered HR in soybean producing Rps1b. Percentage rate of GUS-positive blue spots following co-bombardment of Avr1b with Avr3b or Avr3b^P132A^ compared with EV. Data are the means of four independent experiments. Bars represent standard errors from four independent replicates. **, *t* test *P*<0.01.

In general, cyclophilins preferentially bind to the Glycine-Proline (GP) motif in substrate proteins and catalyze prolyl bond isomerization [41,42]. Sequence analysis identified a potential GP motif (G_131_P_132_) in Avr3b (S8 Fig). To examine whether this putative GP motif is involved in the interaction between Avr3b and GmCYP1, we generated the mutant Avr3b^P132A^ and examined its Nudix hydrolase activity when expressed in *N*. *benthamiana*. Western blot data showed that Avr3b and the mutant Avr3b^P132A^ was expressed in a similar level (S9A and S9B Fig). Proteins extracted from tissues expressing Avr3b^P132A^ showed the same low level of Nudix hydrolase activity as those extracted from *GFP*-expressing tissues, indicating that Avr3b^P132A^ was no longer activated (Fig 6A). We then determined that the interaction between GmCYP1 and Avr3b^P132A^ was significantly weakened in yeast (Fig 6B and S9C Fig), consistent with the notion that Pro132 plays a key role in mediating Avr3b-GmCYP1 interaction. Overall, these results demonstrated that Pro132 of Avr3b is required for Avr3b maturation, probably through direct interaction with GmCYP1.

To further detect the effect of GmCYP1 on the biological functions of Avr3b *in planta*, Avr3b and Avr3b^P132A^ were expressed in *N*. *benthamiana*, and the detached leaves were subsequently challenged with *P*. *capsici*. *P*. *capsici* produced larger lesions on *Avr3b*-expressing leaves than on *Avr3b* ^*P132A*^-expressing leaves (S9D Fig). Quantitative PCR confirmed a higher biomass of *P*. *capsici* in *Avr3b*-expressing leaves, suggesting that Avr3b^P132A^ was unable to promote *P*. *capsici* infection (Fig 6C).

Regarding to the avirulence role of Avr3b during soybean-*P*. *sojae* interaction, we tested whether Avr3b^P132A^ could be recognized by soybean Rps3b plant. Transient expression of Avr3b^P132A^ by bombardment could not induce Rps3b-mediated cell death on soybean leaves (Fig 6D and S9E Fig). Previous studies have shown that Avr3b suppresses cell death induced by interactions between Avr1b and Rps1b [19]; thus, we examined whether Avr3b^P132A^ still retains this virulence function using bombardment. Our data showed that, unlike Avr3b, Avr3b^P132A^ was unable to suppress Avr1b-triggered cell death in soybean (Fig 6E and S9F Fig). These results provide convincing evidence that Pro132 is essential for both avirulence and virulence functions of Avr3b, probably by mediating the interaction of Avr3b with GmCYP1.

### Avr3b specifically interacts with plant cyclophilins, but not with *Phytophthora* CYP homologs

**Fig 7:**
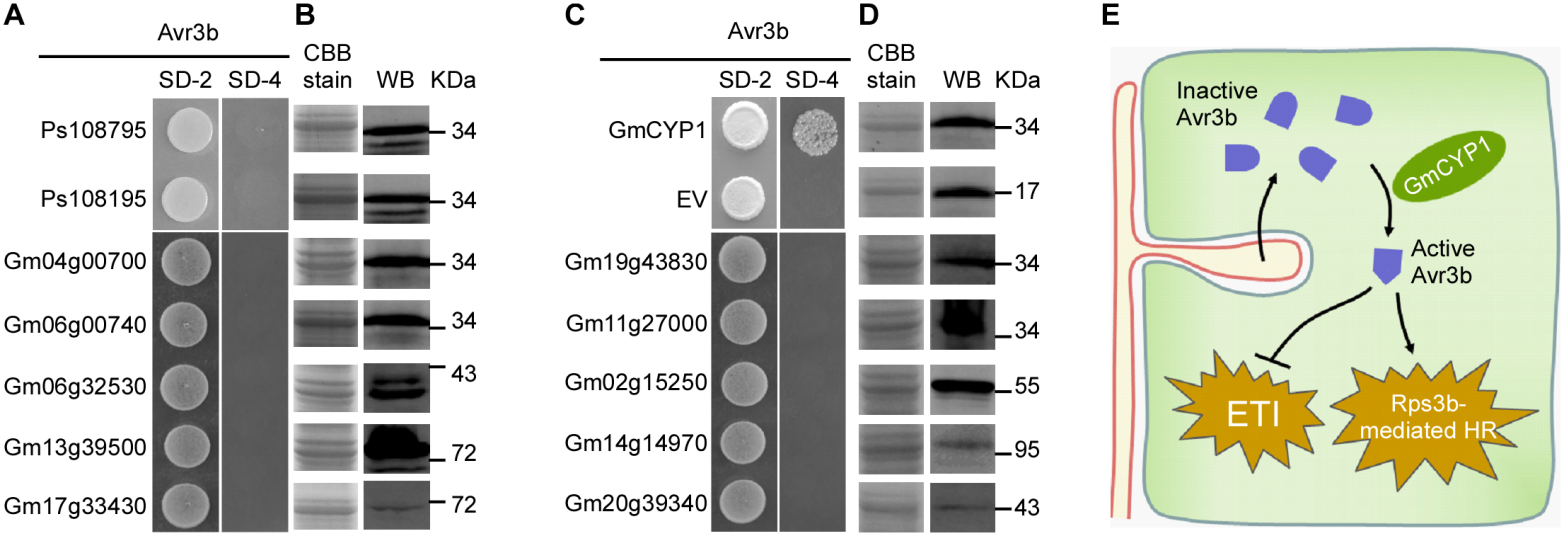
Avr3b specifically interacts with GmCYP1 in soybean. (**A, C**) Avr3b does not interact with other GmCYP1 homologs in soybean or two CYPs of *P*. *sojae*. Selected cyclophilin genes were cloned into pGADT7. Yeast cells were co-transformed with the empty prey vector, pGADT7 or pGADT7 containing one of homologue genes and the bait vector pGBKT7-Avr3b. Yeast transformants were grown on the selective minimal SD medium lacking tryptophan, and leucine (SD-2) or the selective SD medium lacking adenine, tryptophan, histidine, and leucine (SD-4). The plates were photographed 2 days after inoculation. (**B, D**) Western blots showing protein expression of cyclophilin homologs in yeast cells. Anti-HA antibody was used to detect the expression of cyclophilin homologous proteins of soybean and *P*. *sojae*. The same protein gel was stained with Coomassie brilliant blue (CBB) to show loading. WB, western blot. (**E**) A schematic summary illustrating maturation of Avr3b by plant cyclophilin proteins during *Phytophthora* infection. Inactive Avr3b enters plant cell and interacts with plant cyclophilin to gain the Nudix hydrolase activity. Active Avr3b suppresses ETI-associated cell death triggered by Avr1b. However, in the presence of Rps3b, active Avr3b triggers Rps3b-induced HR.

Cyclophilins belong to a large protein family that is present in all cell types of all the organisms studied so far [43]. Previous results showed that Avr3b could not be activated by *P*. *sojae* extracts (Fig 1B), suggesting that Avr3b may not interact with or be processed by *P*. *sojae* cyclophilins. By searching the genomes of soybean and *P*. *sojae*, we found 17 cyclophilin homologues in *P*. *sojae* and 72 cyclophilin homologues in soybean (S2 Table). To test whether *P*. *sojae* cyclophilins could interact with Avr3b, we cloned the genes *Ps108795* and *Ps108195*, which are the most similar to GmCYP1 (79% and 60% identity in full-length amino acid sequences), for Y2H analysis. Our data showed that neither of these *P*. *sojae* cyclophilins could interact with Avr3b in yeast (Fig 7A and 7B). This result supports a hypothesis that Avr3b is likely present as an inactive form before entering the plant cells.

GmCYPs have diverse gene structures and subcellular localizations, indicating that they involve in a large variety of cellular functions [44]. Since GmCYP1 is predicted as a cytosolic cyclophilin [44], we then selected ten soybean cyclophilins including other cytosolic cyclophilins that share high sequence similarity with GmCYP1 for interaction analysis with Avr3b. Interestingly, none of these tested GmCYPs could interact with Avr3b in yeast (Fig 7A–7D). These results suggest that the enzymatic activity of Avr3b is specifically activated by GmCYP1 in soybean.

## Discussion

In this study, we report that a soybean cyclophilin protein GmCYP1 physically interacts with the *Phytophthora* effector Avr3b and activates the Nudix hydrolase activity of Avr3b, which is required for the full virulence of *P*. *sojae*. GmCYP1 possesses the PPIase activity that can be suppressed by the chemical inhibitor CsA. Application of CsA or mutation of catalytic residue Arg62 of GmCYP1 abolished its activating activity on Avr3b, suggesting that maturation of Avr3b in plant cells is dependent on PPIase activity of GmCYP1. Moreover, we identified Pro132 of Avr3b as a key residue to mediate the interaction with GmCYP1. *In planta* expression of Avr3b^P132A^ lost its hydrolase activity and is no longer able to enhance *Phytophthora* infection when expressing in *N*. *benthamiana*. The mutation of Pro132 also abolished Avr3b-induced HR in Rps3b-producing soybean.

Interestingly, we found Avr3b Nudix hydrolase activity can be activated by both *N*. *benthamiana* and soybean extracts, but not *P*. *sojae* extracts. Since the activation of Avr3b by *N*. *benthamiana* extract could be largely impaired by CsA treatment, *N*. *benthamiana* likely provides “helper” molecule(s) possessing PPIase activity for Avr3b activation (S10 Fig). Our experiments further confirmed that two *N*. *benthamiana* cyclophilins, i.e. NbCYP3 and NbCYP4, and one soybean cyclophilin, i.e. GmCYP1, are likely the “helpers” that activate Avr3b in plant cells.

One outstanding question is whether other GmCYP1 homologs can process Avr3b. Our large scale yeast two hybrid assays testing interaction between Avr3b and GmCYP1 homologs suggest that Avr3b specifically interacts with GmCYP1, indicating substrate specificity in cyclophilin homologs in soybean (Fig 7A–7D). This is consistent with previous reports that cyclophilins interact and process different classes of proteins through sequence-specific binding and diverse localization [41,43,45,46].

Previous studies showed that cyclophilins can regulate the protease activity of effector AvrRpt2 from bacteria, conformational change of plant ETI receptor RPM1-interacting protein RIN4, and the binding activity of HCV RNA polymerase NS5B through their PPIase activity [31,33,35]. Our experiments showed that cyclophilins are also associated with the activation of Nudix hydrolase, and that GmCYP1 activates Avr3b in a PPIase-dependent manner. However, whether *cis-trans* isomerization of Avr3b protein actually occurs during *Phytophthora* infection requires further investigation.

To our surprise, the site direct Avr3b^P132A^ mutation not only impaired Avr3b Nudix hydrolase activity but also abolished the Avr3b triggered immunity, suggesting Pro132 is a key residue associated with Rps3b recognition. Interestingly, the investigation of *P*. *sojae* natural populations uncovered only two Avr3b natural alleles, namely the avirulence allele Avr3b^P6497^ and the virulence allele Avr3b^P7076^. Sequence analysis of both alleles revealed a mutation on the Pro132 site in the avirulence sequence into an Alanine at the corresponding position in virulence allele Avr3b^P7076^ (S11 Fig). This natural mutant and the phenotype of the Avr3b^P132A^ mutant constructed in this study suggest that Pro132 is a key residue in Avr3b recognition event. On the other hand, our previous study indicated that the Avr3b^QQQQ^ mutant (Avr3b^R220Q, E221Q, E225Q, E226Q^, a non-functional Nudix hydrolase mutant) could still trigger cell death in soybean, suggesting that Avr3b recognition by the cognate Rps3b receptor is independent on the Nudix hydrolase activity [19]. A possible explanation of these results is that P132 is required for not only the Nudix hydrolyase activity, but also other feature of Avr3b, such as protein conformation changes, which may be required for Avr3b-Rps3b recognition.

In addition to *P*. *sojae*, Avr3b-like effectors are extensively present in other *Phytophthora* species including *P*. *infestans*, *P*. *ramorum* and *P*. *capsici*. Sequence alignment of Avr3b-like effectors indicated that the GP motif containing Pro132 is not conserved among these effectors, although other potential GP motifs are present in a few other effectors (S11 Fig). These data suggested that effector processing by plant cyclophilin proteins might not be required for all the *Phytophthora* Nudix effectors.

It has been widely accepted that pathogen effectors directly target host proteins and manipulate host targets [47]. However, host proteins also manipulate the activity of pathogen effectors. It has been proposed that some host proteins could act as “helpers” to facilitate effector functions, whereas others are “targets” [4]. Previous studies demonstrated that folding of bacterial effector facilitated by host factors might be important to regulate effector functions in host cells during pathogenesis. Previous studies showed that *Arabidopsis* cyclophilin ROC1 is required to activate the cysteine protease activity of the bacterial effector AvrRpt2 [33,34]. Our study demonstrated that a soybean cyclophilin GmCYP1 is responsible for the activation of a *Phytophthora* effector Avr3b as a Nudix hydrolase (Fig 7E). In another word, GmCYP1 is the host helper recruited by Avr3b to become an active virulence protein in plant cells. These results suggest that recruitments of host factors as “helpers” is a common pathogenesis mechanism shared by eukaryotic and prokaryotic pathogens.

## Materials and Methods

### Plant and microbe cultivation

Plants were grown in greenhouse at 22–25°C with a cycle of 16 and 8 hours of high light intensity and darkness, respectively. *P*. *sojae* isolate P6497 and *P*. *capsici* used in this study were grown in 10% vegetable (V8) juice agar medium at 25°C in the dark.

### Yeast two-hybrid screens

*Avr3b* gene without the secretion signal was cloned into the yeast vector pGBKT7 (Clontech). The expression of the BD-Avr3b fusion proteins has been verified by western blots (S12 Fig). A soybean (*Glycine max*, William 82) cDNA library was constructed in the Y2H vector pGADT7 using total RNA extracted from soybean hypocotyl tissues collected 12 and 24 hours after the plants were inoculated with *P*. *sojae* zoospores (Clontech). Approximately 6×10^6^ primary yeast clones (three times coverage of the library) were screened using Avr3b as the baits. Potential yeast transformants containing cDNA clones interacting with Avr3b were selected using the SD/-Trp/-Leu/-His/-Ade selective medium.

### *In vitro* GST pull-down assays

To construct GST-fusion plasmids, Avr3b was inserted into the vector pGEX4T-2 (GE Healthcare Life Science). To construct His-fusion plasmid, GmCYP1 was inserted into the vector pET28a. Pull-down assay was performed using ProFound pull-down GST protein-protein interaction kit (Pierce) according to the manufacturer’s instructions. GST, GST-Avr3b, and His-GmCYP1 was expressed in *E*. *coli* strain BL21 (DE3) respectively. The soluble total proteins were incubated with 50 μl glutathione agarose beads (Invitrogen) for 2 hours at 4°C. The beads were washed five times and then incubated with equal amount of bacterial lysates containing His-GmCYP1 for another hour at 4°C. The beads were washed five times again, and the presence of His-GmCYP1 was detected by western blot using anti-His antibody.

### Nudix hydrolase activity assays

To analysis Nudix hydrolase activity of Avr3b, a general approach was used as described [23]; Reaction mixture (50 μl for each sample) contained 50 mM Tris-HCl, pH 8.5, 1 mM dithiothreitol, 5 mM MgCl_2_, 2 mM substrate (NADH), 2 units of calf alkaline phosphatase, and 2 μg of protein samples. After incubation for 30 min at 37°C, the reaction was stopped by adding 150 μl of 0.5 M H_2_SO_4_, followed by addition of 100 μl of water. For color development, 700 μL of a freshly made mixture containing 600 μl of 0.42% (w/v) ammonium molybdate and 100 μL of 10% (w/v) ascorbic acid was used. The reaction tubes were incubated in 45°C water bath for 20 min for color development and then cooled down to room temperature. The solutions were measured using spectrophotometer (Beckman, USA) at A_820_. The reaction mixture without protein was used as a blank control. For Nudix hydrolase activity assay of total plant protein extract, one *N*. *benthamiana* leaf was infiltrated with FLAG-GFP bacteria in one side and with FLAG-Avr3b or FLAG-Avr3b^P132A^ in another side. A total of 0.2 g of GFP or Avr3b infiltrated leaf tissue was collected at 2 days post infiltration (dpi). For both kinds of protein preparations, the relative hydrolase enzyme activity was calculated as the ratio of A_820_ reading value from sample mixture over control mixture.

### Western blot and co-immunoprecipitation assays

A standard SDS-PAGE protocol was performed for protein separation. Proteins were transferred onto polyvinylidene difluoride (PVDF) membranes using a semi-wet apparatus (Bio-Rad, Hercules, CA,U.S.A.). Then, the membrane was blocked using phosphate-buffered saline (PBS; pH 7.4) with 3% nonfat dry milk for 1 hour at room temperature. Antibodies were added to PBST with 3% nonfat dry milk (PBSTM) at a ratio of 1:4,000 and incubated at 4°C overnight, followed by three washes (10 min each) with PBST. Then, the membrane was incubated with a goat anti-mouse IRDye 800CW (Odyssey,number 926–32210; Li-Cor, Lincoln, NE, U.S.A.) at a ratio of 1:10,000 in PBSTM at room temperature for 2 hours. The membrane was washed four times (10 min each) with PBST, then visualized using Odyssey scanner with excitation at 700 and 800 nm [11].

Avr3b with a N-terminal FLAG-tag was inserted into pGR107, and GmCYP1 was inserted into pBinGFP for expression in *N*. *benthamiana*. The Avr3b^P132A^ and GmCYP1^R62A^ mutants were generated by overlapping PCR using primer described in (S3 Table). FLAG-tagged Avr3b and GFP-tagged GmCYP1 or GFP were co-expressed in *N*. *benthamiana* leaves by agroinfiltration. FLAG-tagged proteins were immunoprecipitated by anti-FLAG M2 affinity gel (Sigma-Aldrich) from total extracts harvested from *N*. *benthamiana* leaves at 2 dpi. The interacting GFP fusion proteins were detected as described above. The expression of FLAG- or GFP-tagged protein in total extract was confirmed by western blot using either anti-FLAG or anti-GFP antibody.

### Recombinant protein expression and purification

Recombinant Avr3b and mutants were overexpressed in *E*. *coli* strain BL21 (DE3). *E*. *coli* clones were grown in LB medium to a density of OD600 = 0.5, and then protein expression was induced overnight at 18°C with 0.4 mM isopropyl β-D-thiogalactopyranoside (IPTG). Cells were lysed in a buffer containing 100 mM Tris-HCl, 150 mM NaCl, 1mM imidazole, 1 mM DTT (pH 8.0). Recombinant GST-Avr3b were affinity purified by Glutathione Sepharose affinity chromatography (GE Healthcare) and eluted from the Glutathione Sepharose with 10 mM reduced glutathione in 100mM HEPES, 150 mM NaCl, 2 mM DTT, and 10% glycerol (pH 7.5). Recombinant CYP1 or mutant proteins were expressed *E*. *coli* BL21(DE3) by pET28a and isolated under native conditions. *E*. *coli* clones were grown in LB to a density of OD600 = 0.6. Protein expression was induced for 6 h at 28°C with 0.5 mM IPTG. Cells were lysed in a buffer containing 10 mM imidazole, 20 mM Tris pH 8.0, 150 mM NaCl. Proteins were purified by Ni+-sepharose affinity chromatography and washed with the same buffer containing 50 mM imidazole. The proteins were eluted with 250 mM imidazole in the same buffer.

### PPIase assays

PPIase assays were conducted essentially as previously described [28]. The mixture of following components were incubated on ice for 10 min: 975 μl HEPES Buffer (35 mM HEPES, 0.015% TritonX-100, pH 8.0), 20 μl of 5 mM N-succinyl-ala-ala-pro-pNa (Baychem), and 8nM of His-GmCYP1 or the GST negative control. Each sample was placed in a spectrophotometer pre-cooled to 8°C. After the addition of 10 mM alpha-chymotrypsin (Sigma), the absorbance at 390 nm was recorded every second for one min at 8°C immediately.

### Avr3b activation reactions

To test the activation of Avr3b proteins by soybean extract, the recombinant Avr3b protein (2 μg) produced and purified from *E*. *coli* was incubated with 100 μg of dialyzed soybean extract, 100 μg of dialyzed *N*. *benthamiana* extract or 100 μg of dialyzed *P*. *sojae* (P6497) mycelium extract respectively in a total volume of 50 μl in buffer containing 50 mM HEPES, 10 mM sodium bisulfite, 10 mM sodium metabisulfite and 1mM DTT (pH 7.5) at 25°C for 15 hours. To assess the activation of Avr3b by GmCYP1, purified recombinant Avr3b (2 μg) was incubated with 1 μg of *E*. *coli* expressed recombinant GmCYP1 protein in a total volume of 50 μl. For inhibition of PPIase activity, 20 μM of cyclosporine A (Sigma) was used in each reaction.

### Virulence assay of *Phytophthora* on *N*. *benthamiana*

4 to 5 week old *N*. *benthamiana* were infiltrated with *Agrobacterium* carrying FLAG-GFP, FLAG-Avr3b or Avr3b mutant. The infiltrated leaves were inoculated with zoospore suspension of *P*. *capsici* 48 hours post *Agro*-infiltration. The total DNA isolated at 36 hours post inoculation. Then primers specific for *P*. *capsici* and *N*. *benthamiana* actin genes were used to quantify the relative biomass of pathogen by quantitative PCR (S3 Table). PCR reactions were performed on an ABI Prism 7500 Fast real-time PCR System (Applied Biosystems, Foster City, CA, U.S.A.).

### Silencing of *GmCYP1* in soybean

The *GmCYP1* hairpin constructs were prepared in a two-step cloning process in pFF19::CHSA plasmid. The chalcone synthase (CHSA) intron was amplified from plasmid pFGC5941. The CHSA intron amplicon was inserted into plant transient expression plasmid pFF19::GUS (replacing GUS with CHSA intron, creating pFF19::CHSA). The PCR-amplified *GmCYP1* cDNA fragments were first insert at outer restriction site (BamHI) and then the inner restriction site (AscI) on either side of the intron sequence. The construct were used by bombardment to induce transiently silencing *GmCYP1* in soybean.

### VIGS of cyclophilins in *N*. *benthamiana*

We used the Tobacco Rattle Virus (TRV)-based VIGS system, which uses bipartite sense RNA1 and RNA2 viruses [48], to silence cyclophilin genes in *N*. *benthamiana*. The *NbCYP3* fragment was amplified by PCR and then cloned into pTRV2 vector. Primer pairs TRVNbCYP3F and TRVNbCYP3R (S3 Table) were used to amplify *NbCYP3*. At four-leaf stage, the *N*. *benthamiana* plants were selected for *Agro*-infiltration. Prior to *Agro*-infiltration, *A*. *tumefaciens* strain GV3101 cells carrying pTRV1 and pTRV2 constructs were collected and resuspended in infiltration buffer (10 mM MgCl2, 150 mM acetosyringone, and 10 mM MES, pH 5.6) and mixed in a 1:1 ratio. Plants were grown for 2 weeks after infiltration. The fully expanded six leaves of the silenced plants were then used for inoculation and quantitative PCR assays. TRV2:*GFP* vector was used as the negative control.

### Particle bombardment assay

*Avr3b*, *Avr3b* ^*P132A*^ and *Avr1b* genes were amplified using specific primers without signal peptide sequences and inserted into plant transient expression plasmid pFF19-GUS (replacing GUS with the test genes). The double-barreled particle bombardment assays was performed on leaves to deliver a parallel control shot in every case that contained GUS DNA plus EV DNA as described [9]. For each paired shot, Specific cell death activity was calculated as the ratio of blue spot numbers for various test gene constructs compared to that of the control. After bombardment, the leaves were incubated for 2 days in darkness at 28°C. The leaves were then stained for 12 h at 37°C using 0.8 mg/mL X-gluc (5-bromo-4-chloro-3-indolyl-β-D-glucuronic acid, cyclohexylammonium salt), 80 mM Na phosphate, pH 7.0, 0.4 mM K_3_Fe(CN)_6_, 0.4 mM K_4_Fe(CN)_6_, 8 mM Na_2_EDTA, and 0.1% (v/v) Triton X-100 and then de-stained in 100% ethanol.

### Bioinformatics

Nudix hydrolase homologs used in this study are acquired as described [19]. The *P*. *sojae* and soybean homolog searches were performed at the Joint Genome Institute database (http://genome.jgi-psf.org) and soybean genome (http://www.phytozome.net/soybean.php). For *N*. *benthamiana* genome searching, the genome sequence was downloaded from the *N*. *benthamiana* database (http://bti.cornell.edu/research/projects/nicotiana-benthamiana/) and a local BLAST search was conducted. Protein domain and motif analyses were conducted using the NCBI conserved domain database (http://www.ncbi.nlm.nih.gov/Structure/cdd/cdd.shtml) and Motif Scan (http://myhits.isb-sib.ch/cgi-bin/motif_scan). Sequence alignment was performed using BioEdit2005.

## Supporting Information

S1 Fig Coomassie blue staining for pull down assay. Whole lysates and the precipitation of GST-Avr3b and GST with the GST-binding resins were examined by Coomassie blue staining to confirm the amounts of GST-Avr3b and GST for Fig 2B. This experiment was repeated three times with similar results.
(TIF)

S2 Fig Cyclophilin genes from soybean, *N*. *benthamiana* and *P*. *sojae* display diversity in sequence. A neighbor-joining tree was constructed based on the conserved cyclophilin domain of 72 soybean (*Glycine max*) cyclophilin genes, 47 *N*. *benthamiana* cyclophilin genes and 17 *P*. *sojae* cyclophilin genes using MEGA5. The representative cyclophilin genes selected to test the interaction with Avr3b were shown. Interaction, cyclophilins interact with Avr3b.
(TIF)

S3 Fig Avr3b interacts with GmCYP1^R62A^. The test of GmCYP1 or GmCYP1 ^R62A^ with Avr3b demonstrates that positive interaction was achieved between Avr3b and GmCYP1 or GmCYP1 ^R62A^ in Y2H system. This experiment was repeated three times with similar results.
(TIF)

S4 Fig Purification of the recombinant proteins. (**A**) SDS-PAGE analysis of GST and GST-Avr3b proteins expressed in *E*.*coli* and purified by GST resins. (**B**) SDS-PAGE analysis of purified His-GmCYP1 and His-GmCYP1^R62A^ produced in *E*. *coli*. The protein bands were visualized using Coomassie blue staining.
(TIF)

S5 Fig Expression of Avr3b with GmCYP1 or GmCYP1^R62A^ in *N*. *benthamiana*. *N*. *benthamiana* leaves were infiltrated with *Agro*-bacterium carrying Avr3b and GmCYP1 or mutant constructs, and total protein was extracted at 48 hpi. Immunoblots were used to detect the expression of Avr3b and GmCYP1 or mutant. This experiment was repeated four times with similar results.
(TIF)

S6 Fig Virus-induced gene silencing (VIGS) of *NbCYP3* and *NbCYP4* in *N*. *benthamiana*. (**A**) Transcript abundance of *NbCYP1*, *NbCYP2*, *NbCYP3*, *NbCYP4*, and *NbCYP5* in *NbCYP3*/*4*-silenced *N*. *benthamiana* leaves, measured by quantitative RT-PCR. Bars represent standard errors from three independent replicates. **, *t* test *P*<0.01. (**B**) *NbCYP3*/*4*-silenced plants did not show marked phenotypic alterations when compared to TRV:*GFP* control plants. This experiment was repeated four times with similar results. (**C**) FLAG-Avr3b and FLAG-RFP (as a negative control) were transiently expressed in *NbCYP3*/*4*-silenced *N*. *benthamiana* leaves. Total proteins were extracted at 48 hpi, then the expression of Avr3b and RFP were confirmed by western blot assay. This experiment was repeated three times with similar results.
(TIF)

S7 Fig Transcription of *GmCYP1* is decreased by *GmCYP1*-RNAi. (**A**) Schematic maps of DNA construct designed for the induction of GmCYP1 silencing. Sense and antisense fragments of *GmCYP1* are separated by the chalcone synthase (CHSA) intron. (**B**)*GmCYP1*-RNAi was transiently expressed by bombardment in soybean leaves. Total RNA of the delivery region were extracted 2 days after bombardment, then Transcript abundance of *GmCYP1*, *Gm04g00700* and *Gm06g00740* were measured by quantitative PCR. Values are means±standard deviations (as error bars) (n = 3). *, *t* test *P*<0.05.
(TIF)

S8 Fig The sequence signature of Avr3b. Predicted signal peptide, RXLR-dEER motif, GP motif, and Nudix motif are shown in grey, orange, blue, and green frames, respectively. The experimentally identified Avr3b-cyclophilin binding residue Proline132 was included in GP motif.
(TIF)

S9 Fig Proline132 of Avr3b plays a critical role in Avr3b activation. (**A, B**) Western blot confirmed the expression of FLAG-GFP and FLAG-Avr3b for Fig 6A and 6C respectively. Total proteins were stained with Ponceau S. (**C**) Avr3b^P132A^ is impaired in its interaction with GmCYP1. Yeast cells transformed with Avr3b^P132A^ and GmCYP1 showed weak interaction than Avr3b and GmCYP1. (**D**) The P132A mutation of Avr3b resulted in reduced virulence activity. FLAG-GFP, FLAG-Avr3b, or FLAG-Avr3b^P132A^ proteins were transiently expressed in *N*. *benthamiana* leaves by *Agro*-infiltration method. These leaves were inoculated with *P*. *capsici* at 48 hpi. Infection of *P*. *capsici* was stained with trypan blue and photographed 36 hours post inoculation. Scale bars, 10mm. (**E**) Avr3b^P132A^ failed to induce Rps3b-mediated cell death in soybean. Avr3b^P132A^ was transiently expressed in soybean cultivars Williams (rps3b) and PRX146-36 (Rps3b) with GUS expression vector by co-bombardment. Scale bars, 3mm. (**F**) Avr3b^P132A^ could not suppress Avr1b-triggered HR in soybean producing Rps1b. The direct comparison of cell death triggered by co-bombardment of Avr1b + empty vector (EV) compared with Avr1b + Avr3b^P132A^ on Rps1b soybean and Williams. These experiments were repeated four times with similar results.
(TIF)

S10 Fig Activation of Avr3b by plant extracts is dependent on PPIase activity present in the extracts. Recombinant Avr3b (2 μg) purified from *E*.*coli* was incubated with 100 μg dialyzed soybean total extract or 100 μg dialyzed *N*. *benthamiana* total extract for 15 hours and then Nudix hydrolase activity of mixtures was analyzed. Nudix hydrolase activity of the mixtures was also examined in the presence of 20 μM CsA comparing with GST. Bars represent standard errors from four independent replicates. *, *t* test P<0.05.
(TIF)

S11 Fig Sequence alignment of Avr3b homologues and characterized GP motif. Predicted *Phytophthora* RXLR effectors (PITG_05846, PITG_06308, PITG_15679, PITG_15732, PrAvh165, PrAvh268, PrAvh281, and Pc102433) are aligned by ClustalW. Avr3b^P6497^ and Avr3b^P7076^ are avirulence and virulence alleles, respectively. GP motifs are shown in frames. The Avr3b natural mutation (Pro132 to Ala130) is highlighted with asterisk.
(TIF)

S12 Fig Avr3b and Avr3b^P132A^ are stable in yeast. Western blot confirmed the expression of Avr3b and Avr3b^P132A^ proteins in yeast cells using anti-Myc antibody.
(TIF)

S1 Table The hydrolytic activities of Avr3b against NADH. (DOC)

S2 Table Cyclophilin homologues in P. sojae and soybean. (XLS)

S3 Table Primers used in this study. (XLS)
