# Supplementary figures and images for: The Activation of Phytophthora Effector Avr3b by Plant Cyclophilin is Required for the Nudix Hydrolase Activity of Avr3b
Source: PLoS Pathog. 2015 Aug 28;11(8):e1005139. doi: 10.1371/journal.ppat.1005139 (PMC4552650; doi:10.1371/journal.ppat.1005139)

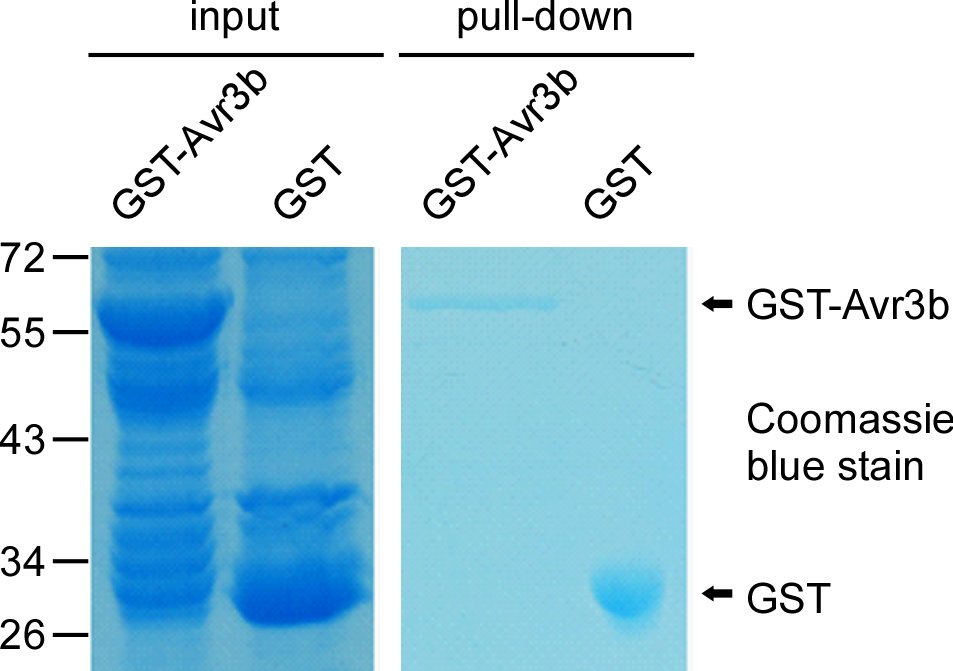

Supplement: S1 Fig — Whole lysates and the precipitation of GST-Avr3b and GST with the GST-binding resins were examined by Coomassie blue staining to confirm the amounts of GST-Avr3b and GST for Fig 2B. This experiment was repeated three times with similar results. (TIF) [file ppat.1005139.s001.tif]

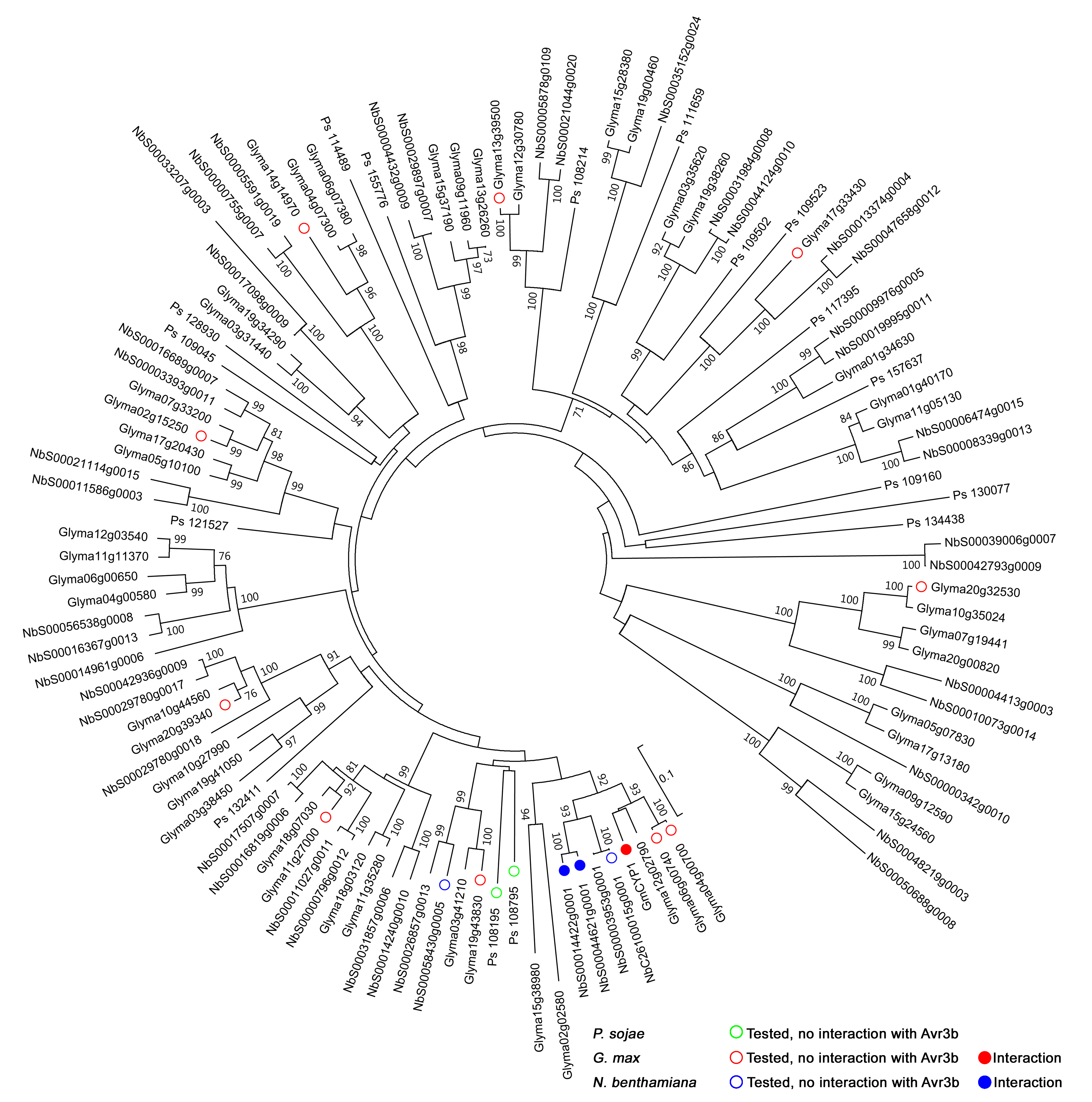

Supplement: S2 Fig — A neighbor-joining tree was constructed based on the conserved cyclophilin domain of 72 soybean (Glycine max) cyclophilin genes, 47 N. benthamiana cyclophilin genes and 17 P. sojae cyclophilin genes using MEGA5. The representative cyclophilin genes selected to test the interaction with Avr3b were shown. Interaction, cyclophilins interact with Avr3b. (TIF) [file ppat.1005139.s002.tif]

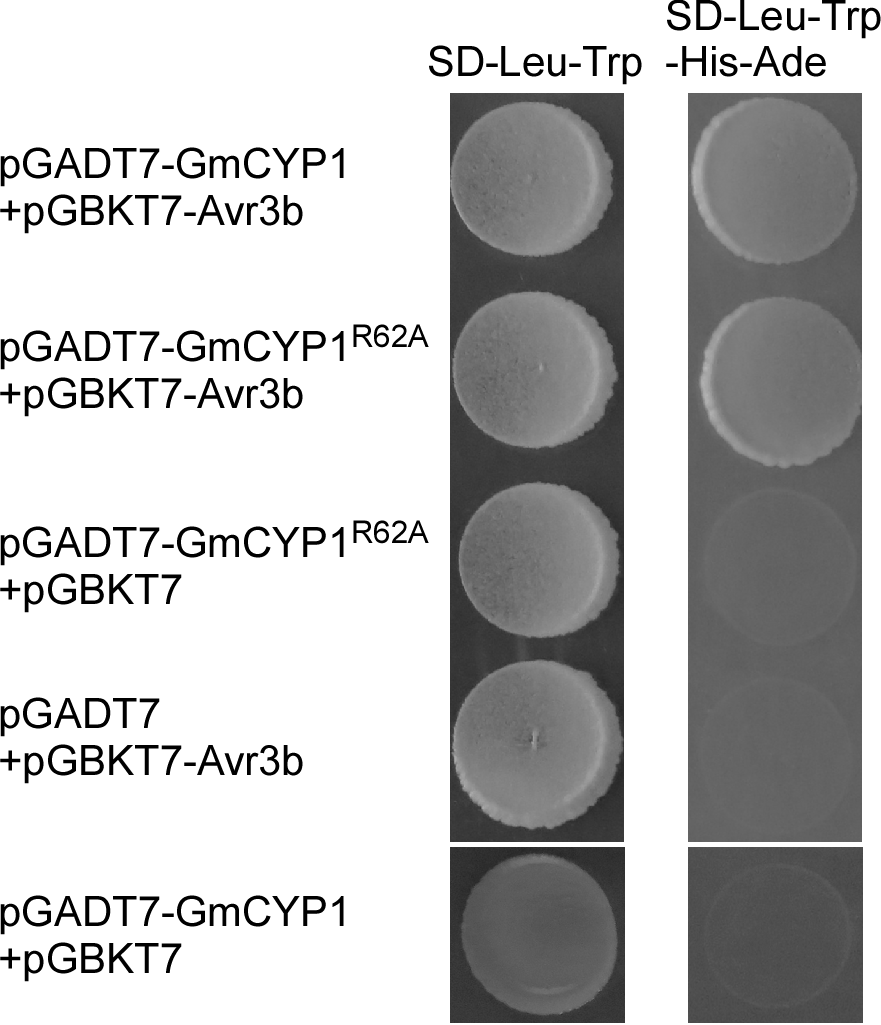

Supplement: S3 Fig — The test of GmCYP1 or GmCYP1 R62A with Avr3b demonstrates that positive interaction was achieved between Avr3b and GmCYP1 or GmCYP1 R62A in Y2H system. This experiment was repeated three times with similar results. (TIF) [file ppat.1005139.s003.tif]

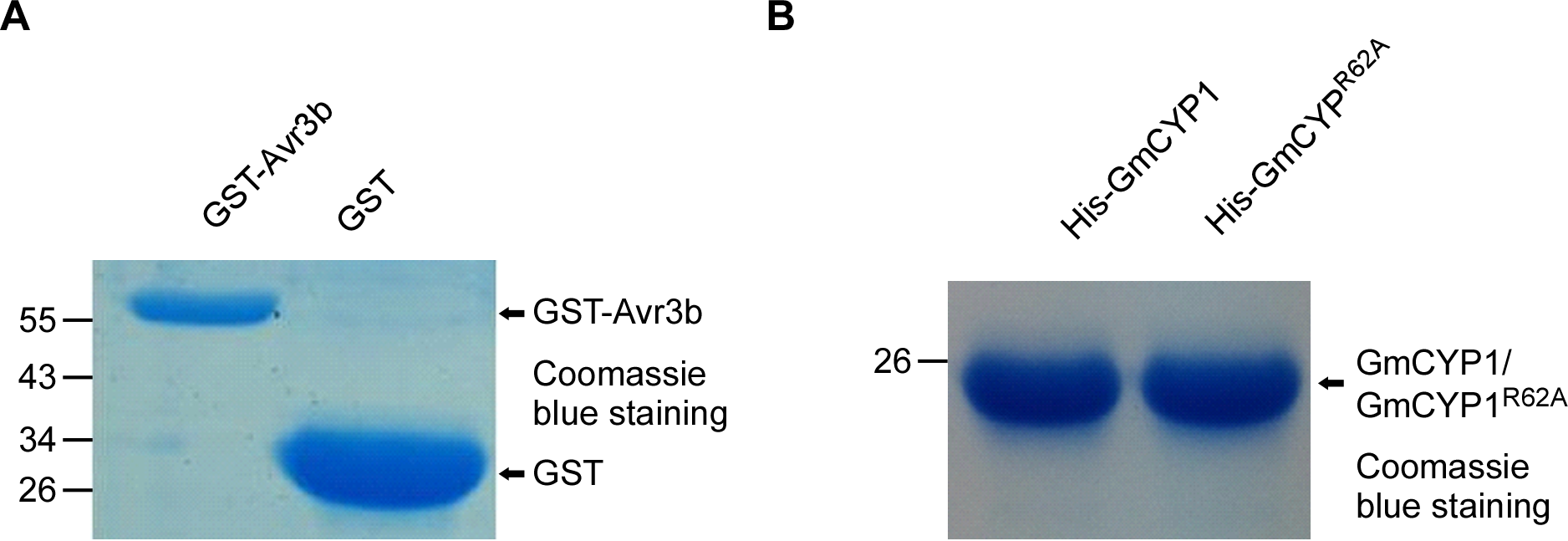

Supplement: S4 Fig — (A) SDS-PAGE analysis of GST and GST-Avr3b proteins expressed in E.coli and purified by GST resins. (B) SDS-PAGE analysis of purified His-GmCYP1 and His-GmCYP1R62A produced in E. coli. The protein bands were visualized using Coomassie blue staining. (TIF) [file ppat.1005139.s004.tif]

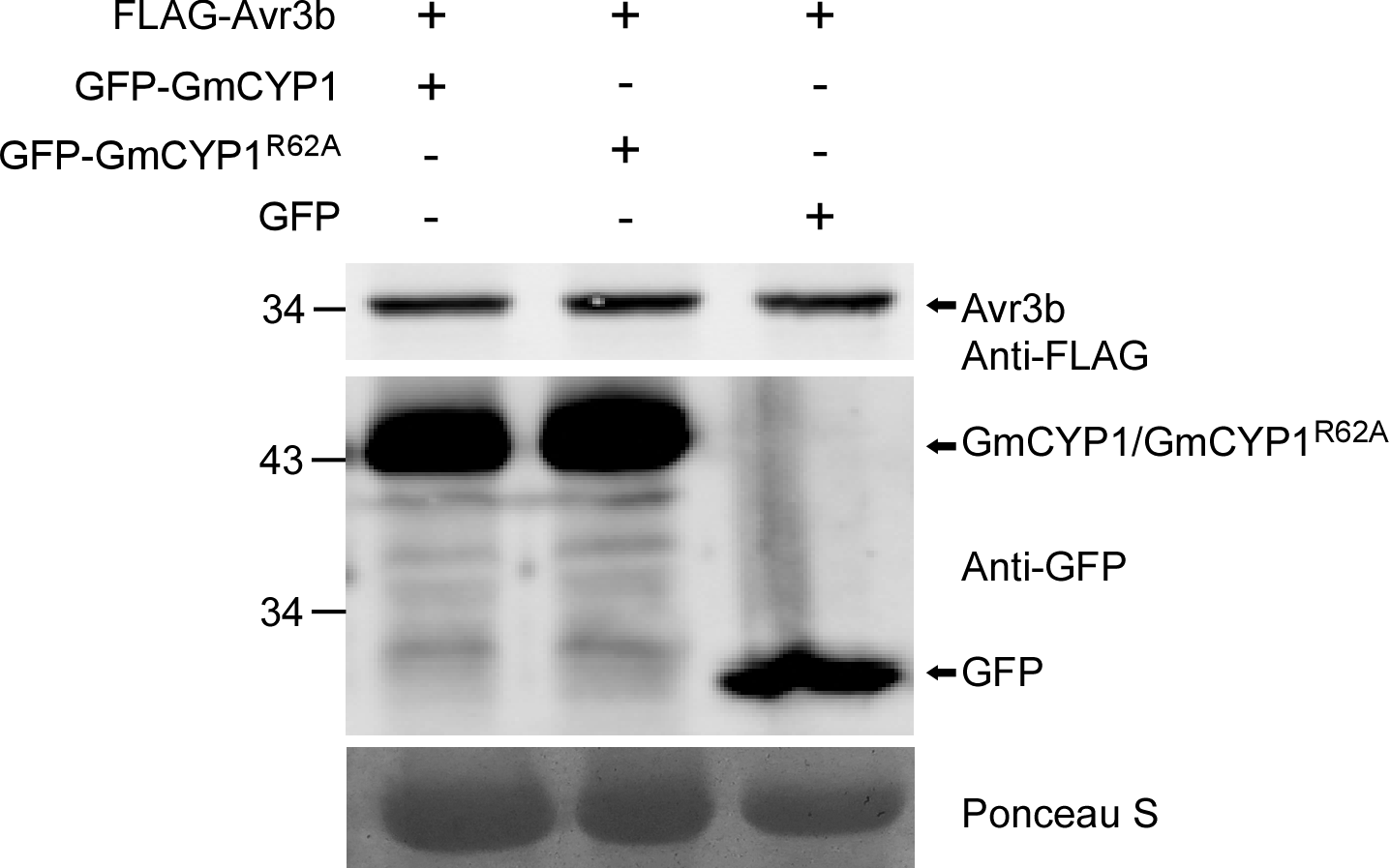

Supplement: S5 Fig — N. benthamiana leaves were infiltrated with Agro-bacterium carrying Avr3b and GmCYP1 or mutant constructs, and total protein was extracted at 48 hpi. Immunoblots were used to detect the expression of Avr3b and GmCYP1 or mutant. This experiment was repeated four times with similar results. (TIF) [file ppat.1005139.s005.tif]

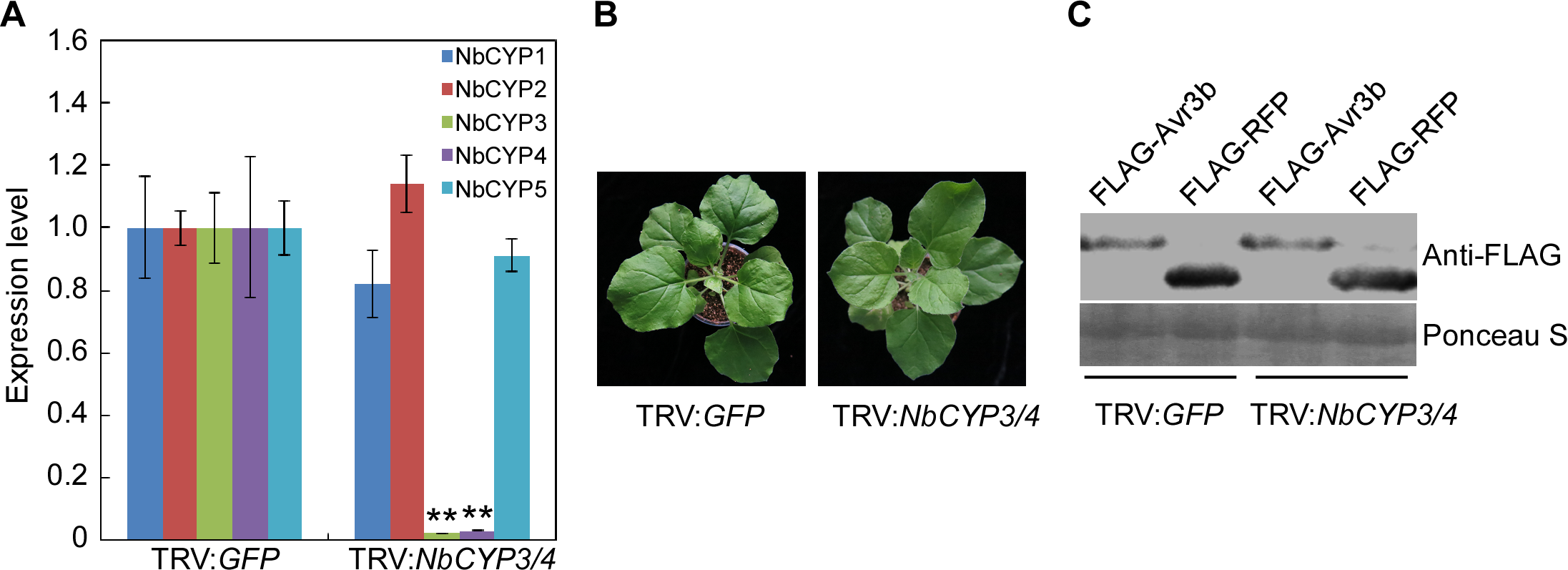

Supplement: S6 Fig — (A) Transcript abundance of NbCYP1, NbCYP2, NbCYP3, NbCYP4, and NbCYP5 in NbCYP3/4-silenced N. benthamiana leaves, measured by quantitative RT-PCR. Bars represent standard errors from three independent replicates. **, t test P<0.01. (B) NbCYP3/4-silenced plants did not show marked phenotypic alterations when compared to TRV:GFP control plants. This experiment was repeated four times with similar results. (C) FLAG-Avr3b and FLAG-RFP (as a negative control) were transiently expressed in NbCYP3/4-silenced N. benthamiana leaves. Total proteins were extracted at 48 hpi, then the expression of Avr3b and RFP were confirmed by western blot assay. This experiment was repeated three times with similar results. (TIF) [file ppat.1005139.s006.tif]

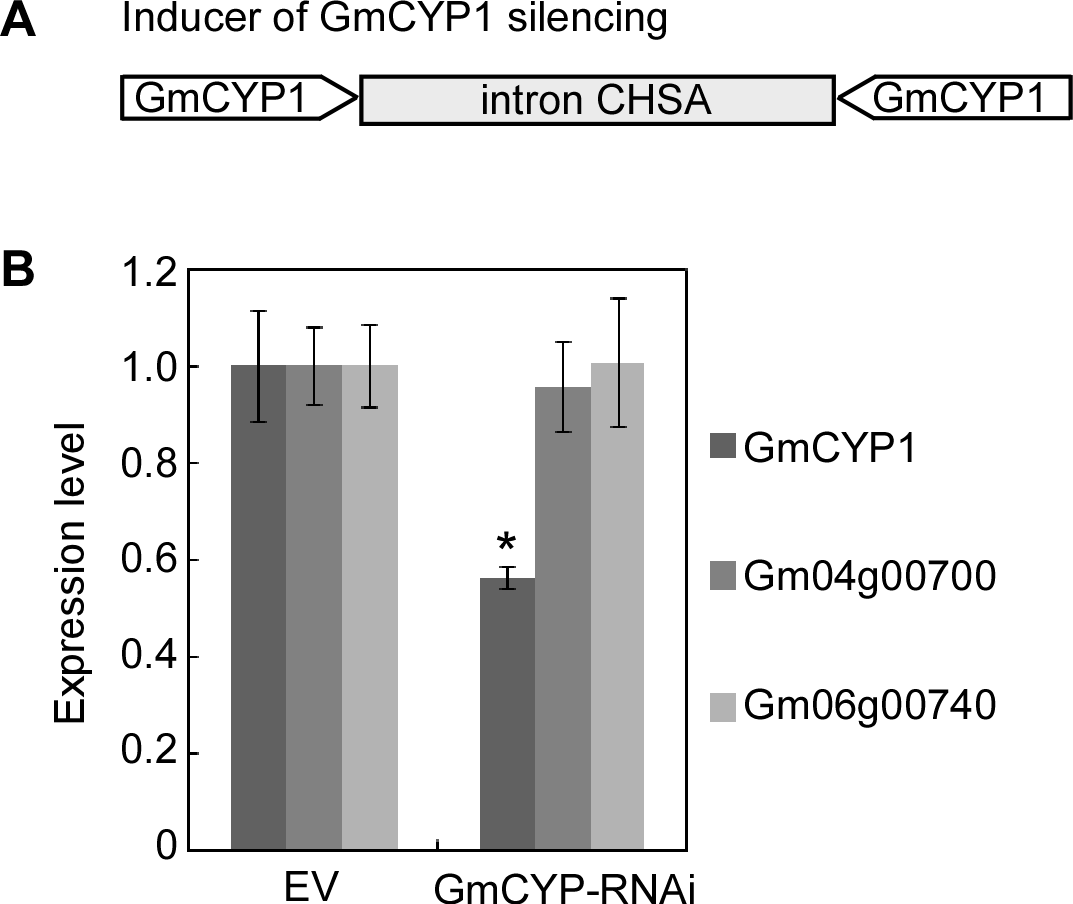

Supplement: S7 Fig — (A) Schematic maps of DNA construct designed for the induction of GmCYP1 silencing. Sense and antisense fragments of GmCYP1 are separated by the chalcone synthase (CHSA) intron. (B)GmCYP1-RNAi was transiently expressed by bombardment in soybean leaves. Total RNA of the delivery region were extracted 2 days after bombardment, then Transcript abundance of GmCYP1, Gm04g00700 and Gm06g00740 were measured by quantitative PCR. Values are means±standard deviations (as error bars) (n = 3). *, t test P<0.05. (TIF) [file ppat.1005139.s007.tif]

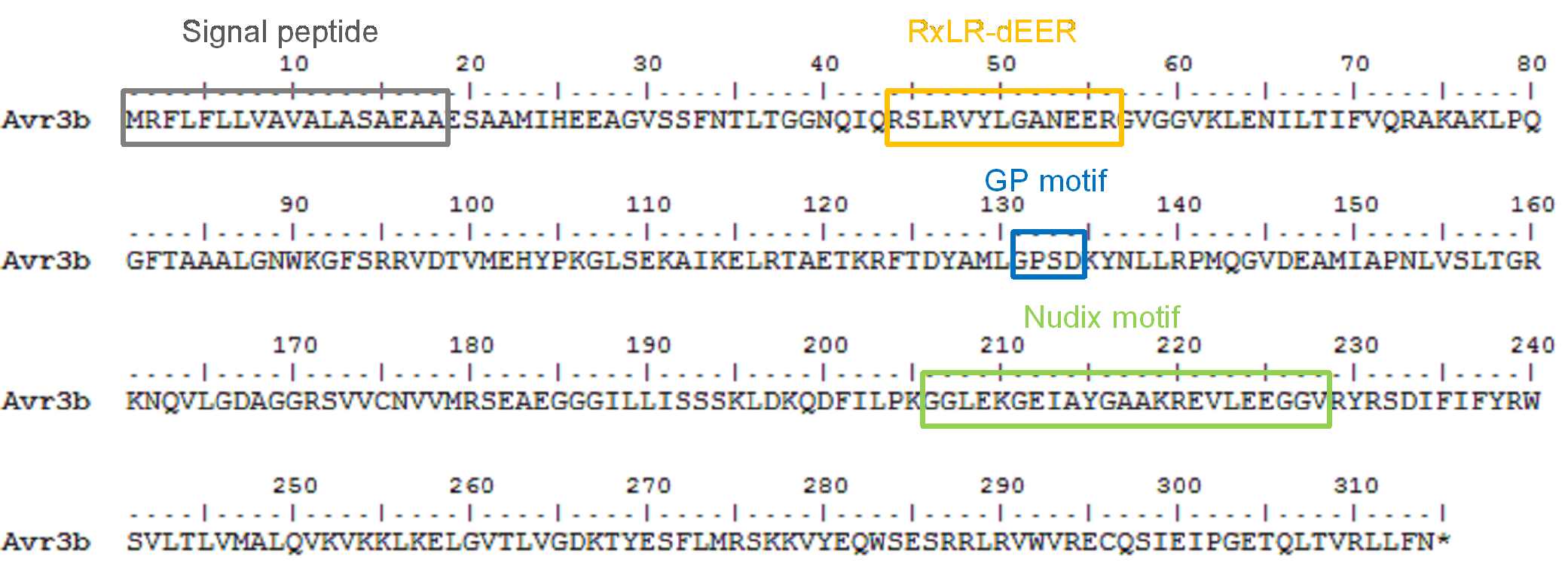

Supplement: S8 Fig — Predicted signal peptide, RXLR-dEER motif, GP motif, and Nudix motif are shown in grey, orange, blue, and green frames, respectively. The experimentally identified Avr3b-cyclophilin binding residue Proline132 was included in GP motif. (TIF) [file ppat.1005139.s008.tif]

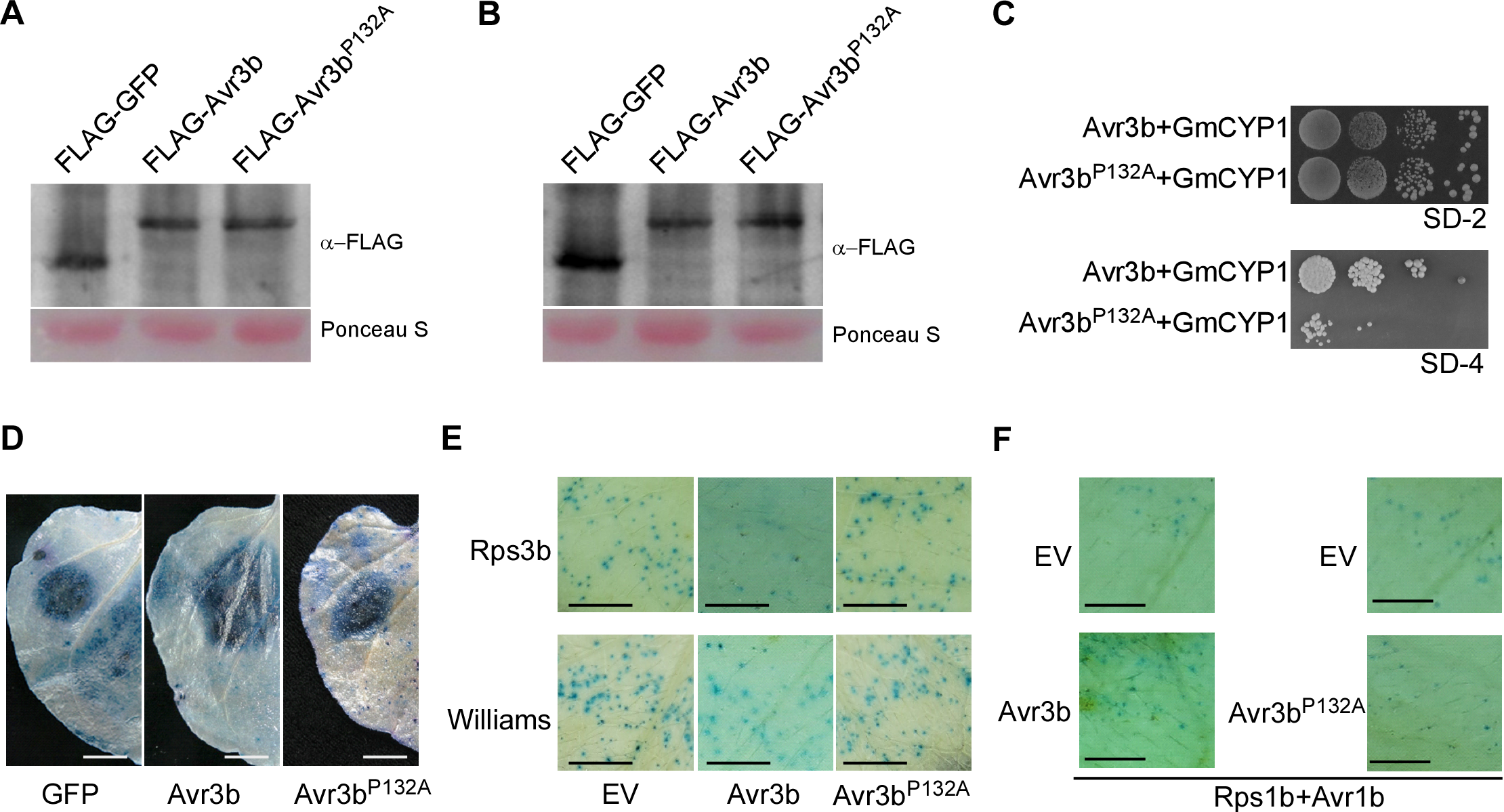

Supplement: S9 Fig — (A, B) Western blot confirmed the expression of FLAG-GFP and FLAG-Avr3b for Fig 6A and 6C respectively. Total proteins were stained with Ponceau S. (C) Avr3bP132A is impaired in its interaction with GmCYP1. Yeast cells transformed with Avr3bP132A and GmCYP1 showed weak interaction than Avr3b and GmCYP1. (D) The P132A mutation of Avr3b resulted in reduced virulence activity. FLAG-GFP, FLAG-Avr3b, or FLAG-Avr3bP132A proteins were transiently expressed in N. benthamiana leaves by Agro-infiltration method. These leaves were inoculated with P. capsici at 48 hpi. Infection of P. capsici was stained with trypan blue and photographed 36 hours post inoculation. Scale bars, 10mm. (E) Avr3bP132A failed to induce Rps3b-mediated cell death in soybean. Avr3bP132A was transiently expressed in soybean cultivars Williams (rps3b) and PRX146-36 (Rps3b) with GUS expression vector by co-bombardment. Scale bars, 3mm. (F) Avr3bP132A could not suppress Avr1b-triggered HR in soybean producing Rps1b. The direct comparison of cell death triggered by co-bombardment of Avr1b + empty vector (EV) compared with Avr1b + Avr3bP132A on Rps1b soybean and Williams. These experiments were repeated four times with similar results. (TIF) [file ppat.1005139.s009.tif]

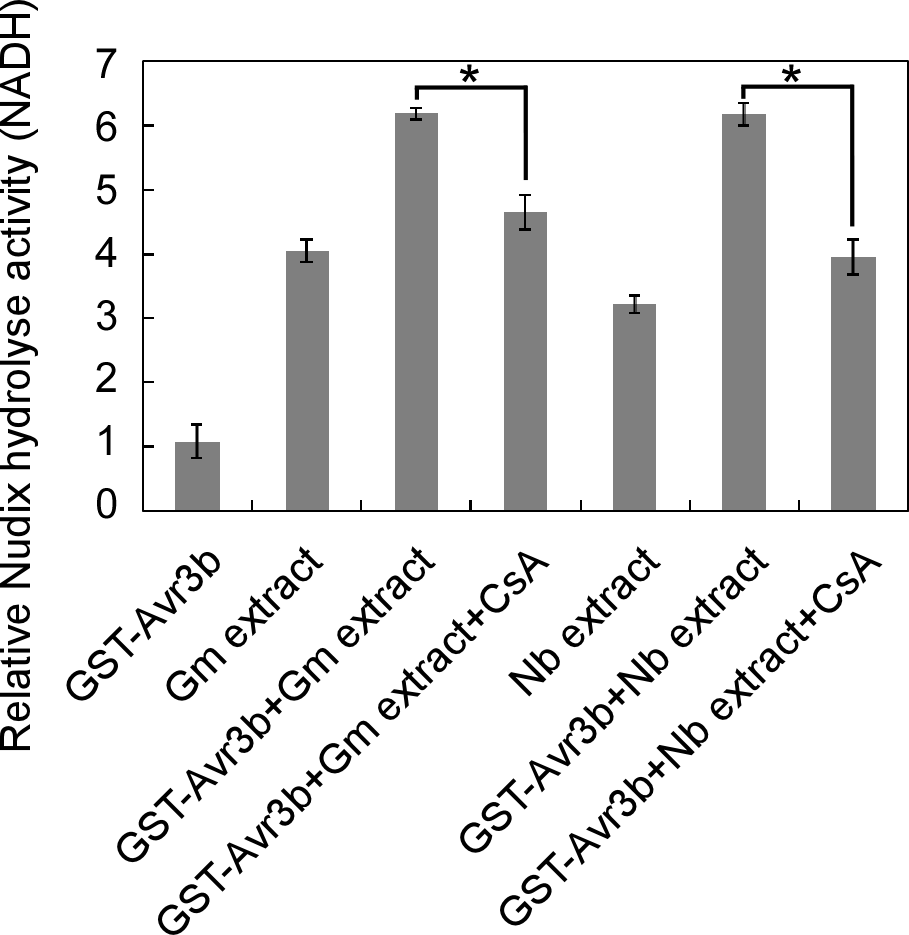

Supplement: S10 Fig — Recombinant Avr3b (2 μg) purified from E.coli was incubated with 100 μg dialyzed soybean total extract or 100 μg dialyzed N. benthamiana total extract for 15 hours and then Nudix hydrolase activity of mixtures was analyzed. Nudix hydrolase activity of the mixtures was also examined in the presence of 20 μM CsA comparing with GST. Bars represent standard errors from four independent replicates. *, t test P<0.05. (TIF) [file ppat.1005139.s010.tif]

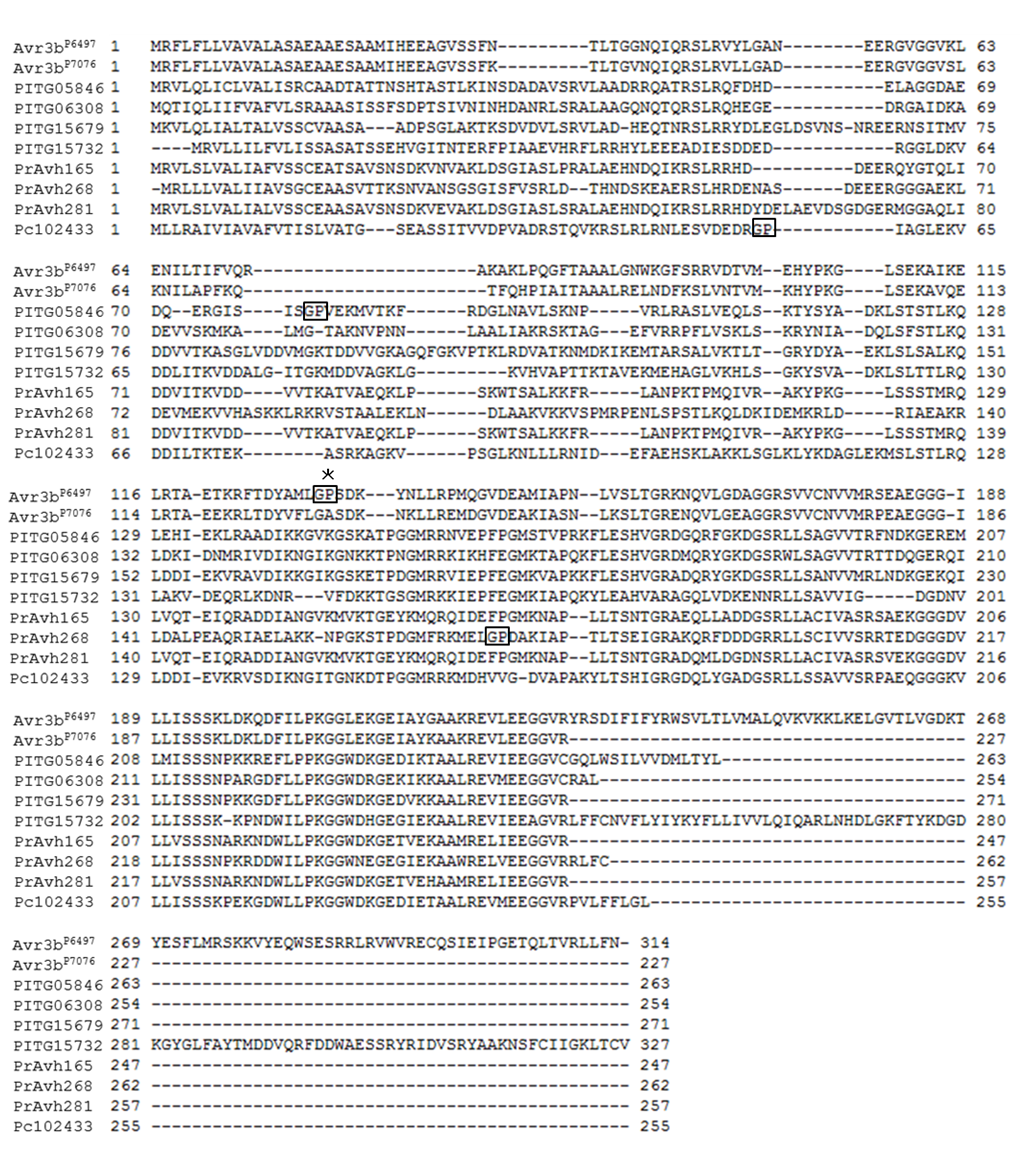

Supplement: S11 Fig — Predicted Phytophthora RXLR effectors (PITG_05846, PITG_06308, PITG_15679, PITG_15732, PrAvh165, PrAvh268, PrAvh281, and Pc102433) are aligned by ClustalW. Avr3bP6497 and Avr3bP7076 are avirulence and virulence alleles, respectively. GP motifs are shown in frames. The Avr3b natural mutation (Pro132 to Ala130) is highlighted with asterisk. (TIF) [file ppat.1005139.s011.tif]

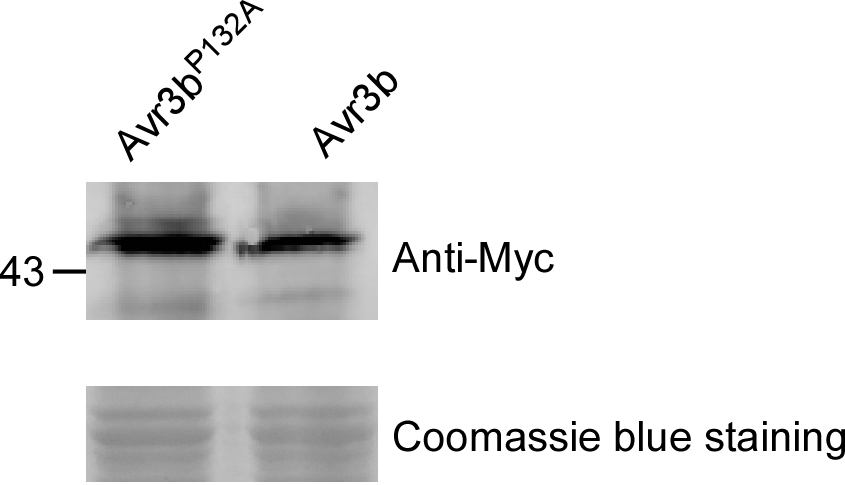

Supplement: S12 Fig — Western blot confirmed the expression of Avr3b and Avr3bP132A proteins in yeast cells using anti-Myc antibody. (TIF) [file ppat.1005139.s012.tif]
