# Supplementary material for: The Activation of Phytophthora Effector Avr3b by Plant Cyclophilin is Required for the Nudix Hydrolase Activity of Avr3b
Source: PLoS Pathog. 2015 Aug 28;11(8):e1005139. doi: 10.1371/journal.ppat.1005139 (PMC4552650; doi:10.1371/journal.ppat.1005139)
Supplement: S1 Table — (DOC) [file ppat.1005139.s013.doc]

**S1 Table. The Nudix hydrolytic activities of both pure Avr3b protein and plant protein extract**

| **Immunoprecipitates^a^** | **Avr3b relative activity^b^** |
| --- | --- |
| Avr3b | 1.05±0.11 |
| Avr3b+GmCYP1 | 2.73±0.20^c^ |
| Avr3b+GmCYP1+CsA | 1.44±0.12^d^ |
| Avr3b+GmCYP1^R62A^ | 1.10±0.19 |
|  |  |
|  |  |
| Total extract^e^ | Avr3b relative activity^f^ |
| Avr3b+GFP | 1.89±0.27 |
| Avr3b+GmCYP1 | 3.90±0.33^g^ |
| Avr3b+GmCYP1^R62A^ | 1.83±0.40 |

**a:** Proteins were produced from *E. coli*. Nicotinamide adenine dinucleotide reduced form (NADH) was used in Nudix hydrolase activity assays.

**b:** The hydrolase activity (A_820_ reading) of Avr3b samples relative to the activity of control samples (GST).

**c:** Significantly different than the Avr3b, (*p*<0.01, student’s *t* test).

**d:** Significantly different than the Avr3b, (*p*<0.05, student’s *t* test).

**e:** Total protein extract from *N. benthamiana* tissue transiently expressing *Avr3b* with *GFP*, *GmCYP1*, or *GmCYP1^R62A^*.

**f:** The hydrolase activity (A_820_ reading) of samples (Avr3b co-expressed with GFP, GmCYP1, or GmCYP1^R62A^) relative to the activity of control samples (from *GFP*-expressing tissue).

**g:** Significantly different than the Avr3b+GFP, (*p*<0.01, student’s *t* test).
